# Distinct Immunological Landscapes Characterize Inherited and Sporadic Mismatch Repair Deficient Endometrial Cancer

**DOI:** 10.3389/fimmu.2019.03023

**Published:** 2020-01-09

**Authors:** Neal C. Ramchander, Neil A. J. Ryan, Thomas D. J. Walker, Lauren Harries, James Bolton, Tjalling Bosse, D. G. Evans, Emma J. Crosbie

**Affiliations:** ^1^University of Manchester Medical School, Manchester, United Kingdom; ^2^Manchester Royal Infirmary, Manchester University NHS Foundation Trust, Manchester, United Kingdom; ^3^Division of Cancer Sciences, Faculty of Biology, Medicine and Health, University of Manchester, Manchester, United Kingdom; ^4^Division of Evolution and Genomic Medicine, Faculty of Biology, Medicine and Health, St. Mary's Hospital, University of Manchester, Manchester, United Kingdom; ^5^Department of Histopathology, Manchester University NHS Foundation Trust, Manchester, United Kingdom; ^6^Department of Pathology, Leiden University Medical Center, Leiden, Netherlands; ^7^Manchester Centre for Genomic Medicine, Manchester Academic Health Science Centre, St. Mary's Hospital, Manchester University NHS Foundation Trust, Manchester, United Kingdom; ^8^Department of Obstetrics and Gynaecology, Manchester Academic Health Science Centre, St. Mary's Hospital, Manchester University NHS Foundation Trust, Manchester, United Kingdom

**Keywords:** Lynch Syndrome, endometrial cancer, mismatch repair, microsatellite instability, immune microenvironment, PD-1, immune checkpoint, predictive modeling

## Abstract

Around 30% of endometrial cancers (EC) are mismatch repair (MMR) deficient, mostly as a consequence of mutations acquired during tumorigenesis, but a significant minority is caused by Lynch syndrome (LS). This inherited cancer predisposition syndrome primes an anti-cancer immune response, even in healthy carriers. We sought to explore the intra-tumoral immunological differences between genetically confirmed LS-associated MMR-deficient (MMRd), sporadic MMR-deficient, and MMR-proficient (MMRp) EC. Endometrial tumors from women with known LS were identified (*n* = 25). Comparator tumors were recruited prospectively and underwent microsatellite instability (MSI) testing, immunohistochemistry (IHC) for MMR expression and *MLH1* methylation testing. Those found to have *MLH1* hypermethylation formed the sporadic MMR-deficient group (*n* = 33). Those found to be mismatch repair proficient and microsatellite stable formed the MMR-proficient group (*n* = 35). A fully automated monoplex IHC panel was performed on sequential formalin-fixed paraffin-embedded tumor sections to identify CD3+, CD8+, CD45RO+, FoxP3+, and PD-1+ immune cells, and PD-L1 expression by tumor/immune cells. Two independent observers quantified immune marker expression at the tumor center and invasive margin. Mean and overall compartmental T-cell counts generated standard (binary: Low/High) and higher resolution (quaternary: 0–25, 25–50, 50–75, 75–100%) immune scores, which were used as explanatory features in neural network, support vector machine, and discriminant predictive modeling. Overall T-cell counts were significantly different between the three cohorts: CD3+ (*p* = <0.0001), CD8+ (*p* = <0.0001), CD45RO+ (<0.0001), FoxP3+ (*p* = <0.0001), and PD1+ (*p* = <0.0001), with LS-associated MMR-deficient tumors having highest infiltrations. There were significant differences in CD8+ (*p* = 0.02), CD45RO+ (*p* = 0.007), and PD-1+ (*p* = 0.005) T-cell counts at the invasive margin between LS-associated and sporadic MMR-deficient tumors, but not between sporadic MMR-deficient and MMR-proficient tumors. Predictive modeling could accurately determine MMR status based on CD8+ T-cell counts within the tumor center alone. This study shows that LS-associated and sporadic MMR-deficient EC are distinct immunological entities, which has important implications for treatment and prognosis.

## Introduction

In the UK, endometrial cancer (EC) is the fourth most common malignancy in women, and its incidence is rising (1). Most EC is sporadic but a significant minority is caused by inherited pathogenic variants in one of the mismatch repair (MMR) genes, *MLH1, MSH2, MSH6*, or *PMS2*, known as Lynch syndrome (LS) (2). A dysfunctional MMR system fails to identify and correct DNA replication errors, potentiating malignant transformation (3–5). The acquisition of point mutations within microsatellite regions is known as microsatellite instability (MSI), and is a hallmark of MMR deficient tumors (6, 7).

MMR deficiency is a feature of LS (LS-associated MMRd), but also arises sporadically (sporadic MMRd) as a consequence of hypermethylation of the *MLH1* promoter region (8). Microsatellite instable EC tumors are characterized by dense immune infiltrates (9, 10) due to the translation of neoantigens, called frameshift peptides (FSP), derived from non-synonymous point and frameshift mutations in protein-coding DNA (11, 12). Some of these FSPs are processed into major histocompatibility complex (MHC) compatible FSP-epitopes that are recognized by T-cells. In colorectal cancer (CRC), MSI is associated with upregulation of genes relating to anti-tumor cytotoxicity, heavy cytotoxic T-cell infiltration, and a favorable prognosis (13). Galon and colleagues have shown that the “Immunoscore,” which quantifies the T-cell density at the invasive margin (IM) and tumor center (CT), is strongly prognostic (13–22); in fact, in CRC the Immunoscore is of superior prognostic value to MSI status (13). This may indicate that the survival benefit of the MSI phenotype is derived from the immunological response to these tumors. The tumor immune microenvironment is dynamic; iterative selection pressures favor the survival of cancer cell clones that have evolved immune evasion mechanisms (23, 24). These include upregulation of the PD-1/PD-L1 axis leading to receptor-mediated inhibition of PD-1 expressing cytotoxic T-cells (25). In addition, the upregulation of FoxP3+ regulatory T-Cells (Tregs) has been described in MSI tumors (26); together, these mechanisms prevent the priming of activated, cytotoxic T cells (27).

The mechanisms of immune evasion are potential therapeutic targets. Monoclonal antibodies against the PD-1 receptor reverse immune quiescence by overcoming tumor-based PD-L1 inactivation of anti-tumor cytotoxic T-cells (27). The success of PD-1 immune checkpoint inhibitors in MMR-deficient tumors is well-established in CRC (28, 29), and expected in EC (30). However, the impact of such therapies in LS is yet to be described. Recent data suggest that the immunological signatures of sporadic and LS-associated MMR-deficient EC are different; in a study where germline confirmation of LS status was not possible, “likely”–LS-associated EC were characterized by a higher cytotoxic T-cell density than sporadic MMR-deficient EC (9).

The aim of this study was to confirm and build on previous work by directly comparing the immunological landscape of proven LS-associated MMR-deficient, sporadic MMR-deficient and MMR-proficient EC. We hypothesized that distinctive immunological profiles characterizing each tumor subgroup would enable their discrimination by machine learning algorithms. Thus, we employed conventional automated immunohistochemistry (IHC) with subsequent manual counts of immune cells. These data then informed probabilistic modeling to identify immunological differences between the cohorts.

## Methods

### Ethics and Regulatory Approvals

The study was approved by the North Lancaster Research Ethics Committee (ref: 15/NW/0733 and 16/NW/0164) and sponsored by the University of Manchester, UK. All participants gave written, informed consent for their data and tumor tissue to be used for research.

### Participants and Tumor Collection

LS-associated tumors were collected from women with germline proven LS, identified through Lynch Syndrome UK, a patient support group, and the LS clinical database held at the Manchester Centre for Genomic Medicine at St. Mary's Hospital (31). Formalin fixed paraffin embedded (FFPE) tumor blocks were obtained from the treating institution and a representative haematoxylin and eosin (H&E) stained slide underwent contemporaneous review by two consultant gynecological pathologists to confirm endometrial site, histological subtype, grade, extent of myometrial invasion, lymphovascular space invasion (LVSI), and to confirm loss of expression of the corresponding MMR protein by IHC.

**Figure 1:**
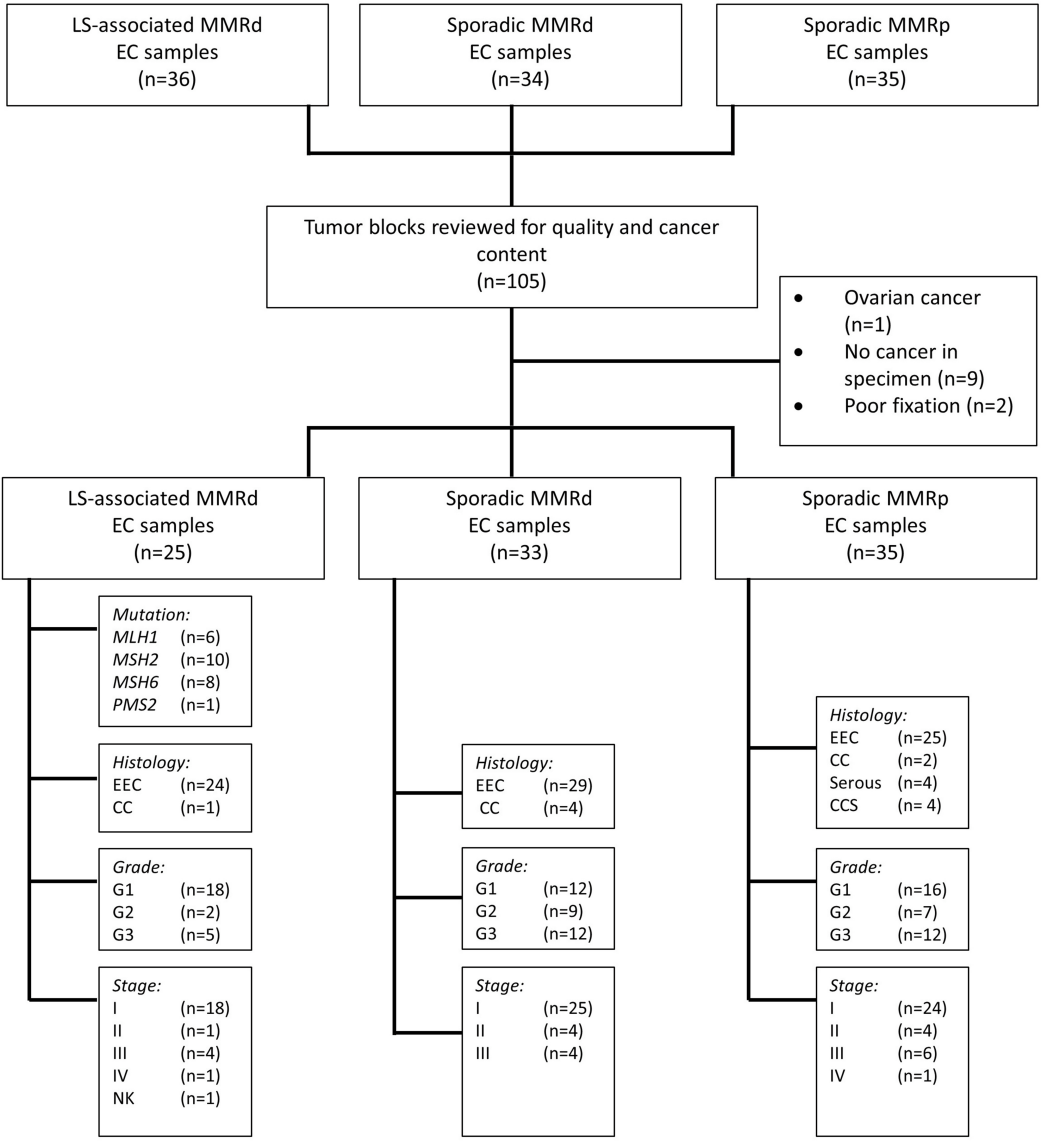
Study flow schema. EEC, Endometrioid Endometrial Cancer; CC, Clear Cell Endometrial Cancer; CCS, Carcinosarcoma of the uterus; G, Grade; NK, Not known; MMRd, mismatch repair deficient; MMRp, mismatch repair proficient.

The sporadic MMR-deficient and MMR-proficient tumors were collected prospectively from women undergoing primary treatment for EC at St. Mary's Hospital, a large Gynecological Cancer Centre in the North West of England, between 2015 and 2017 (Figure 1). Consecutive tumors were tested for MSI and MMR protein expression (using IHC). Those MSI EC tumors found to have MLH1 or PMS2 loss on IHC were further tested for *MLH1* promoter methylation; tumors with *MLH1* hypermethylation comprised the sporadic MMR-deficient cohort. Tumors that expressed all MMR proteins and showed microsatellite stability (MSS) comprised the MMR-proficient cohort. Sporadic MMR-deficient (*n* = 35) and MMR-proficient (*n* = 35) ECs were selected for inclusion by matching them to the LS-associated (*n* = 35) ECs according to their pathological parameters, principally, tumor histology, stage and grade. Those with poor tissue fixation or insufficient EC content were excluded from the study. Representative tumor blocks were either from the hysterectomy specimen (*n* = 66) or diagnostic biopsy specimen (*n* = 27).

### Immunohistochemistry

All IHC analysis was performed using the fully automated Bond Max or Bond Rx (Leica Biosystems, Wetzlar, Germany) platforms. Automated IHC to detect MMR protein expression was performed according to standardized clinical protocols (Supplementary Appendix 1). Automated IHC protocols to detect CD3, CD8, CD45RO, FoxP3, PD-1, and PD-L1 expression were optimized using the Ventana BenchMark ULTRA IHC/ISH Staining Module (Ventana Co., Tucson, AZ, United States) and the OptiView, 3,3' diaminobenzidine version 5 detection system (Ventana Co.) (Supplementary Appendixes 2, 3). Following protocol optimization, six sequential tissue sections per tumor were cut and stained. The order in which each of the six tissue sections were stained with the six primary antibodies was standardized across tumors. All tissue sections were stained within a week of sectioning.

### Microsatellite Instability Analysis

Microsatellite instability was analyzed according to standardized clinical protocols using the MSI analysis system v1.2 (Promega, Wisconsin, United states) (32). Briefly, fluorescent-labeled primers were used to co-amplify seven markers, including five mononucleotide-repeat markers (BAT-25, BAT-26, NR-21, NR-24, and MONO-27), and two control pentanucleotide-repeat markers (Penta C and Penta D). Matching sequence sizes between EC and lymphocytic tissue from the same patient in at least four mononucleotide microsatellite loci was considered microsatellite stable (MSS). A discrepancy in more than two mononucleotide microsatellite loci was considered MSI (depicted in Supplementary Appendix 1).

### Methylation Analysis

Reflex *MLH1* methylation testing was performed on MLH1 and/or PMS2 deficient tumors (33). Briefly, DNA extracted from 10 μm tumor scrapings underwent sodium bisulfite conversion using the Epitect Plus FFPE kit (Qiagen, UK), according to manufacturer's instructions. The purified and eluted product underwent amplification. The sequence of the *MLH1* region of interest is shown in Supplementary Appendix 1. Amplicons were sequenced using the Pyrosequencer (PSQ 96MA). Two independent blinded scientists interpreted the Pyrograms. A significant result was recorded if >10% methylation was recoded at each cytosine in >66% of the triplicates.

### Immune Scoring

MMR IHC slides were light microscope-scored as per standard clinical care (34). An MMR proficient result was recorded if all four MMR proteins were seen in >80% of the tumor. Staining intensity observed in the internal controls (immunopositive lymphocytes, non-malignant stroma, or benign endometrium) was used to inform the interpretation of MMR protein expression.

Slides stained for CD3, CD8, CD45RO, PD-1, and FoxP3 were digitalized using a Pannoramic Scan II (3D Histech, Budapest, Hungary) and interpreted using CaseViewer v2.1 (3D Histech). Investigators were blinded to MMR status and clinicopathological characteristics at the time of scoring. Immune cells with positive membranous staining (CD3, CD8, CD45RO, PD-1) or nuclear staining (FoxP3) were counted within 200 μm^2^ regions of interest (ROI) at the tumor center (CT) and invasive margin (IM; where the tumor abuts normal stromal tissue) using the cell counter feature on CaseViewer. The ROI were initially selected using CD3 immunostained slides (Supplementary Appendix 4). Eight 200 μm^2^ ROI with the highest density of CD3+ T-cells were selected at the CT (x4) and the IM (x4). At the IM, ROI were selected to include 50% healthy tissue abutting the tumor, and 50% tumor tissue. At the CT, ROI were selected to include representative portions of stroma and malignant epithelial glands relative to the tumor grade; higher grade tumors by definition have higher gland:stroma ratios. Intra-stromal and intra-epithelial immune cells were counted. For diagnostic biopsy specimens, only CT (x4) ROI were defined, as the IM could not be discerned. A second observer validated that the selected ROI were (1) the densest CD3+ T-cell areas on the slide and (2) the IM ROI included equal proportions of healthy/tumor tissue. In a handful of cases, ROI were re-selected after discussion. Next, ROI were selected for scoring CD8+ T-cells, CD45RO+ T-cells, FoxP3+ regulatory T-cells, and PD-1+ immune-cells on sequential slides by geographically matching the ROI selected to score CD3+ T-cells. In total, 3,180 200 μm^2^ ROI were manually scored using CaseViewer cell marker overlays. Permanent records of all ROI and the manually placed scoring overlays exist. Tumor expression of PD-L1 was scored with a light microscope using a semi-quantitative scoring method as described elsewhere (35). Membranous expression of PD-L1 on tumor cells was considered positive tumor staining. Staining intensity was scored as weak (1+), moderate (2+), and strong (3+). Greater than 5% tumor positivity at ≥2+ intensity was considered positive tumor expression of PD-L1. Immune cell expression of PD-L1 was also recorded as absent, focal, or diffuse. Samples with focal or diffuse staining were recorded as positive. A subset of immunostained slides (*n* = 100) was second scored, and a Cronbach's alpha of 0.92 was achieved.

### Statistical Analysis and Probabilistic Modeling

#### Software and Data Tidying

Statistical analyses were conducted in R 3.4.0 × 64/RStudio IDE 1.0.143. Packages additional to base R were: caret, e1071, pROC, MASS, ggplot2, plotly, heatmaply, viridis, RColorBrewer with relevant dependencies.

#### Descriptive Variance Analysis

IHC count normality distributions were inspected by histogram and quantile-quantile (QQ) plots of variables and linear modeling residuals (Supplementary Appendix 5). Heteroscedasticity was assessed by Levene's, Bartlett and Fligner-Killeen. Parametric variance was inspected by one-way ANOVA with Tukey's HSD *post-hoc* using the Benjamini Hochberg (FDR) correction. Non-parametric data were analyzed by Kruskal-Wallis with *post-hoc* Dunn test and Benjamini-Hochberg. Comparisons with Games-Howell assessed effects of heteroscedasticity and unequal sample sizes as necessary. Statistical adjustments ensured observer variance or diagnostic vs. hysterectomy sampling did not impact downstream modeling. Machine learning schemas are depicted in Supplementary Appendixes 6, 7.

#### Immune Score Calculations

**Figure 2:**
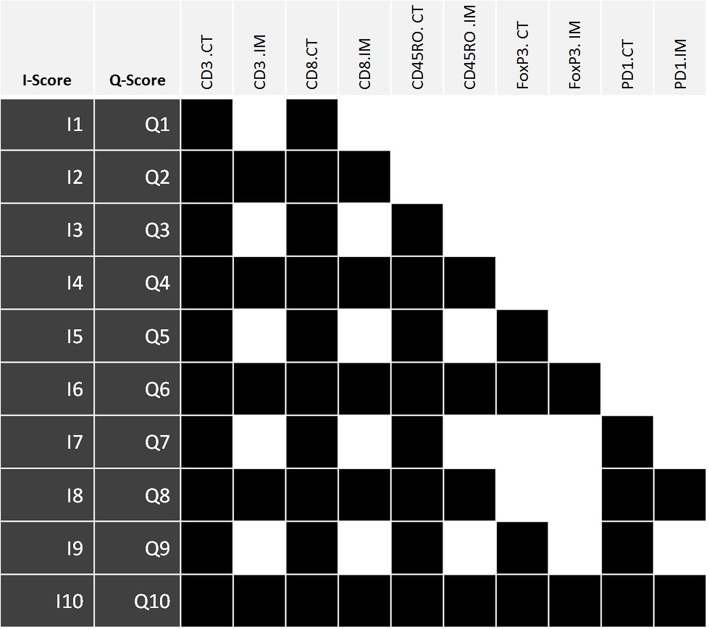
The components of immune scores I1:I10 and Q1:Q10. Each immune score is defined by the density of a set panel of immune markers (CD3, CD8, CD45RO, FoxP3, PD-1) at the tumor center (CT) +/– invasive margin (IM) relative to the corresponding densities across all tumors. The I-scores define each lymphocyte population using the median threshold methodology (0:Low, 1:High), while the Q-scores are defined using quartile ranges (0: 0–25%, 1: 25–50%, 2: 50–75%, 3: 75–100%). Therefore, for any given I-score, the corresponding Q-score presents a higher resolution scoring system. Highlighted boxes indicate inclusion of that particular lymphocyte population within an immune score. I-score, binary immune score; Q-score, quaternary immune score; CT, Tumor Center; IM, Invasive margin.

Ordinal value transformation defined median thresholds for CD3^CT^, CD3^IM^, CD8^CT^, and CD8^IM^. The median threshold methodology is concordant with the original “Immunoscore” defined by Galon et al. (16). These formed immune scores I1 and I2. Subsequent scores I3:I10 incorporated additional markers beyond CD3 and CD8 (these additional markers were CD45RO, PD-1 and FoxP3; immune score compositions are outlined in Figure 2). ROI of each patient's lymphocyte population per CT and IM location were summed and recoded as either 0 (low) or 1 (high) with respect to the cohort's median. Populated binary substitution matrices were used to generate immune scores I1:I10, which represent ten combinations of lymphocyte markers from different tumor regions.

Original I1:I10 immune score resolutions were increased via a quaternary substitution approach wherein summed ROI of a lymphocyte population were recoded as either 0 (0–25%), 1 (25–50%), 2 (50–75%), or 3 (75–100%) with respect to quartile ranges and used to generate quaternary immune scores (Q-scores) Q1:Q10. Specific combinations of lymphocyte markers for Q1:Q10 immune scores exactly match those of the I1:I10 feature selection. Thus, for any given I-score, the corresponding Q-score presents a higher resolution scoring system.

#### Statistical Modeling—Immune Scores

Immune scores I1:I10 and Q1:Q10 were assessed for their predictive classification power of a tumor's molecular profile. ***Three-class predictions***(LS-associated MMR-deficient vs. sporadic MMR-deficient vs. MMR-proficient): A 75 observation equal-class dataset was randomly subsetted from observation data using molecular profile as the response variable. ***Two-class predictions***([LS-associated MMR-deficient + sporadic MMR-deficient] vs. MMR-proficient): The molecular profile was recoded into a new two-class response variable of “MMR-deficient” or “MMR-proficient,” and a 70 observation equal-class dataset was randomly subsetted from observation data.

Predictive modeling details (partitioning, model construction, method type, model performance, model tuning and overfitting) are provided in Supplementary Appendix 6. Predictive power was assessed by confusion matrices and receiver operating characteristic (ROC) curves with associated area under curve (AUC) values [including multiclass generalization of AUC by Hand and Till (36)].

#### Statistical Modeling—Biomarker Counts

Predictive modeling was extended to accommodate summed raw counts of each lymphocyte location as explanatory variables during model construction in order to compare efficacies with the 20 single-feature immune scores (I1:I10 and Q1:Q10). Identical three class and two class data subsets were used as before for model training and testing. For ***three-class*** ***predictions***(LS-associated MMR-deficient vs. sporadic MMR-deficient vs. MMR-proficient), raw counts of each lymphocyte location were coordinated into 32-feature selection cohorts wherein each cohort contained between four and 11 lymphocyte populations (Supplementary Appendix 7, Figure S10). For ***two-class predictions***([LS-associated MMR-deficient + sporadic MMR-deficient] vs. MMR-proficient): eight feature selection cohorts were constructed from the raw counts of CD3 and/or CD8 lymphocytes at the CT and/or IM (Supplementary Appendix 7, Figure S11). Model train-control, tuning, and predictive power assessment proceeded as previously described.

## Results

### Cohort Characteristics

In total, 93 EC tumors were included in this study: LS-associated (*n* = 25), sporadic MMR-deficient (*n* = 33), and MMR-proficient (*n* = 35) (Figure 1). Patient demographics and tumor characteristics are shown in Table 1. There was no significant difference between the three cohorts with respect to histological subtype, grade or stage of disease, or whether the tumor sample derived from the hysterectomy specimen or the diagnostic biopsy. The LS-associated cohort was significantly younger at diagnosis than the sporadic MMR-deficient and MMR-proficient cohorts (mean age 52, 67, and 62 years, respectively, ANOVA *p* = 0.0001); however, there was no significant difference between the sporadic MMR-deficient and MMR-proficient cohorts (*p* = 0.14 Student *t*-test). The LS-associated cohort included *MLH1* (*n* = 6), *MSH2* (*n* = 10), *MSH6* (*n* = 8), and *PMS2* (*n* = 1) pathogenic variant carriers.

**Table 1:** Patient demographics and tumor characteristics.

	**LS-associated MMRd (*n* = 25)**	**Sporadic MMRd (*n* = 33)**	**Sporadic MMRp (*n* = 35)**	
	***n* (%)**	***n* (%)**	***n* (%)**	***p*-value**
**SPECIMEN TYPE**				
Biopsy	5 (20)	12 (36)	10 (29)	(*p =* 0.4)
Hysterectomy	20 (80)	21 (64)	25 (71)	
Mean age at diagnosis	52	67	62	(*p =* <0.0001)
**STAGE**				
I–II	19 (76)	29 (88)	28 (80)	(*p =* 0.5)
III–IV	6 (24)	4 (12)	7 (20)	
**GRADE**				
G1–2	20 (80)	21 (64)	23 (66)	(*p =* 0.4)
G3	5 (20)	12 (36)	12 (34)	
**HISTOLOGY**				
Endometrioid	24 (96)	29 (88)	25 (71)	(*p =* 0.1)
Non-endometrioid	1 (4)	4 (12)	10 (29)	
High grade serous	0	0	4	
Clear cell	1	4	2	
Carcinosarcoma	0	0	4^#^	

*LS-associated MMRd, Lynch Syndrome-associated mismatch repair deficient; Sporadic MMRd, Sporadic mismatch repair deficient; MMRp, mismatch repair proficient*.

# *Epithelial component is endometrioid*.

### Comparing T-Cell Counts Across Cohorts

**Figure 3:**
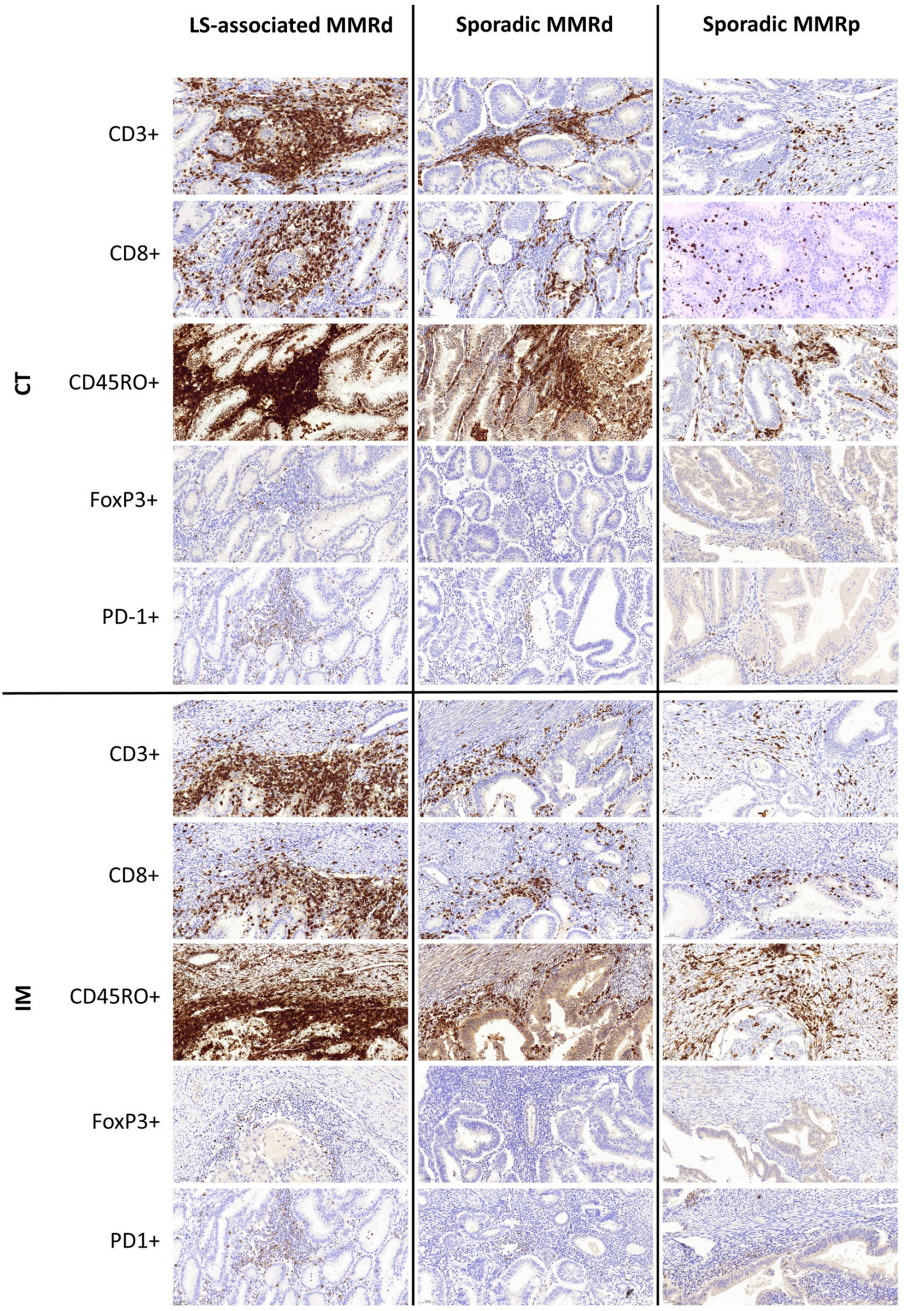
Representative immunohistochemistry images of immune densities across the three molecular cohorts at the Tumor Center (CT) and Invasive Margin (IM).

**Figure 4:**
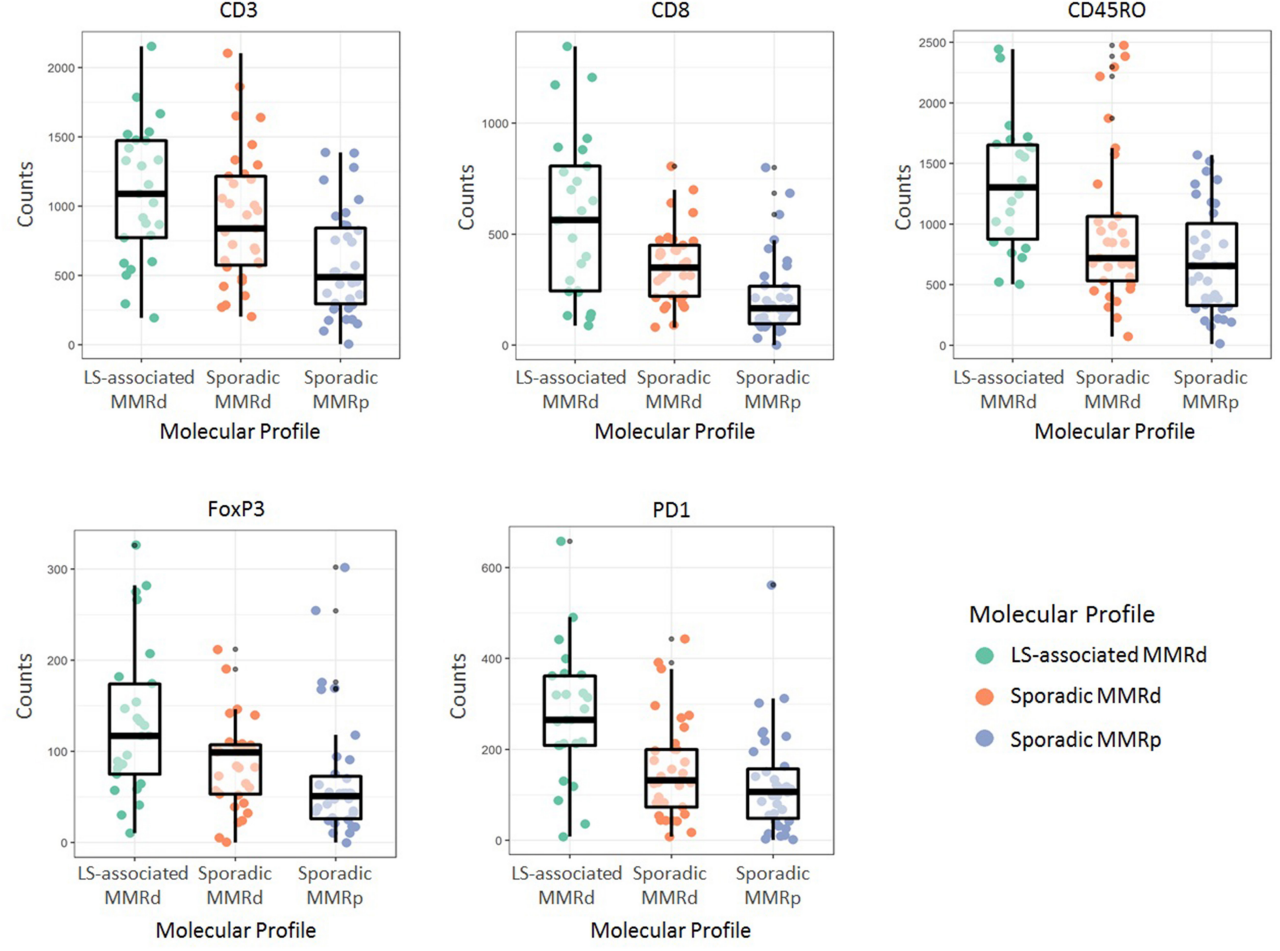
Immune cell counts as per their molecular profile. LS-associated MMRd, Lynch Syndrome-associated mismatch repair deficient; Sporadic MMRd, Sporadic mismatch repair deficient; MMRp, mismatch repair proficient.

Immune cell counts across molecular group and tumor compartment are summarized in Table 2. There was a significant difference in CD3+ (*p* = <0.0001), CD8+ (*p* = <0.0001), CD45RO+ (<0.0001), FoxP3+ (*p* = <0.0001), and PD1+ (*p* = <0.0001) T-cell counts between the three molecular cohorts (Table 3 and Figures 3, 4). PD-L1 immune cell expression was also significantly different across the three cohorts (*p* = 0.04), with the MMR-proficient cohort contributing 63% of the negative results. PD-L1 tumor expression at ≥5% was not significantly different between the three cohorts (*p* = 0.48). Differences in CD8+, CD45RO+, FoxP3+ and PD1+ T-cell counts were significant in two-class analysis between LS-associated vs. sporadic MMR-deficient tumors, however, there was no difference (*p* = 0.99) in PD-L1 expression between these two groups. Subgroup analyses with non-endometrioid tumors excluded, and adjusting for age differences between the cohorts, did not affect the results.

**Table 2:** Immune cell counts by molecular group and location within the tumor.

**Molecular subgroup**	**Marker**	**Mean (SE)**		
		**Overall**	**Tumor center**	**Invasive margin**
LS-associated MMRd	CD3	1,088 (98)	590 (53)	386 (52)
Sporadic MMRd	CD3	918 (83)	617 (59)	241 (39)
MMRp	CD3	581 (63)	380 (40)	157 (32)
LS-associated MMRd	CD8	583 (72)	291 (33)	287 (45)
Sporadic MMRd	CD8	357 (30)	233 (22)	116 (20)
MMRp	CD8	222 (31)	140 (18)	80 (17)
LS-associated MMRd	CD45RO	1,165 (133)	597 (82)	548 (81)
Sporadic MMRd	CD45RO	968 (113)	653 (83)	296 (52)
MMRp	CD45RO	693 (75)	450 (41)	236 (47)
LS-associated MMRd	FoxP3	133 (17)	72 (8)	61 (12)
Sporadic MMRd	FoxP3	86 (8)	58 (7)	28 (4)
MMRp	FoxP3	69 (12)	46 (9)	23 (6)
LS-associated MMRd	PD-1	275 (29)	156 (18)	118 (20)
Sporadic MMRd	PD-1	157 (19)	108 (13)	49 (10)
MMRp	PD-1	125 (19)	83 (13)	42 (10)

*LS-associated MMRd, Lynch Syndrome-associated mismatch repair deficient; Sporadic MMRd, Sporadic mismatch repair deficient; MMRp, mismatch repair proficient*.

**Table 3:** The difference in immune-profile expression by molecular group and location within the tumor.

**Molecular subgroup**	**Marker**	***p*****-value**		
		**Overall**	**Tumor center**	**Invasive margin**
Overall (LS-associated MMRd vs. Sporadic MMRd vs. MMRp)	CD3	**<0.0001**	**<0.0001**	**<0.0001**
LS-associated vs. sporadic MMRd	CD3	0.09	0.4	0.054
LS-associated vs. MMRp	CD3	**0.0001**	**0.002**	**<0.0001**
Sporadic MMRd vs. MMRp	CD3	**0.0024**	**0.003**	**0.006**
Overall (LS-associated MMRd vs. Sporadic MMRd vs. MMRp)	CD8	**<0.0001**	**<0.0001**	**<0.0001**
LS-associated vs. sporadic MMRd	CD8	**0.04**	0.2	**0.02**
LS-associated vs. MMRp	CD8	**<0.0001**	**<0.0001**	**<0.0001**
Sporadic MMRd vs. MMRp	CD8	**0.001**	**0.001**	**0.004**
Overall (LS-associated MMRd vs. Sporadic MMRd vs. MMRp)	CD45RO	**<0.0001**	0.08	**<0.0001**
LS-associated vs. sporadic MMRd	CD45RO	**0.008**	0.2	**0.0007**
LS-associated vs. MMRp	CD45RO	**<0.0001**	**0.04**	**0.0004**
Sporadic MMRd vs. MMRp	CD45RO	**0.04**	0.1	0.1
Overall (LS-associated MMRd vs. Sporadic MMRd vs. MMRp)	FoxP3	**<0.0001**	**<0.0001**	**<0.0001**
LS-associated vs. sporadic MMRd	FoxP3	**0.04**	0.08	0.08
LS-associated vs. MMRp	FoxP3	**0.0002**	**0.0009**	**0.0006**
Sporadic MMRd vs. MMRp	FoxP3	**0.02**	**0.02**	**0.03**
Overall (LS-associated MMRd vs. Sporadic MMRd vs. MMRp)	PD-1	**<0.0001**	**<0.0001**	**<0.0001**
LS-associated vs. sporadic MMRd	PD-1	**0.002**	**0.04**	**0.005**
LS-associated vs. MMRp	PD-1	**<0.0001**	**0.0007**	**0.0001**
Sporadic MMRd vs. MMRp	PD-1	0.09	0.05	0.1

*LS-associated MMRd, Lynch Syndrome-associated mismatch repair deficient; Sporadic MMRd, Sporadic mismatch repair deficient; MMRp, mismatch repair proficient. Unless otherwise stated p values represent Kruskal Wallis with Dunn post hoc, controlled for by Benjamini-Hochberg FDR*.

*All statistically significant results (p < 0.05) are in bold*.

### Comparing Immune Scores Across Cohorts

**Figure 5:**
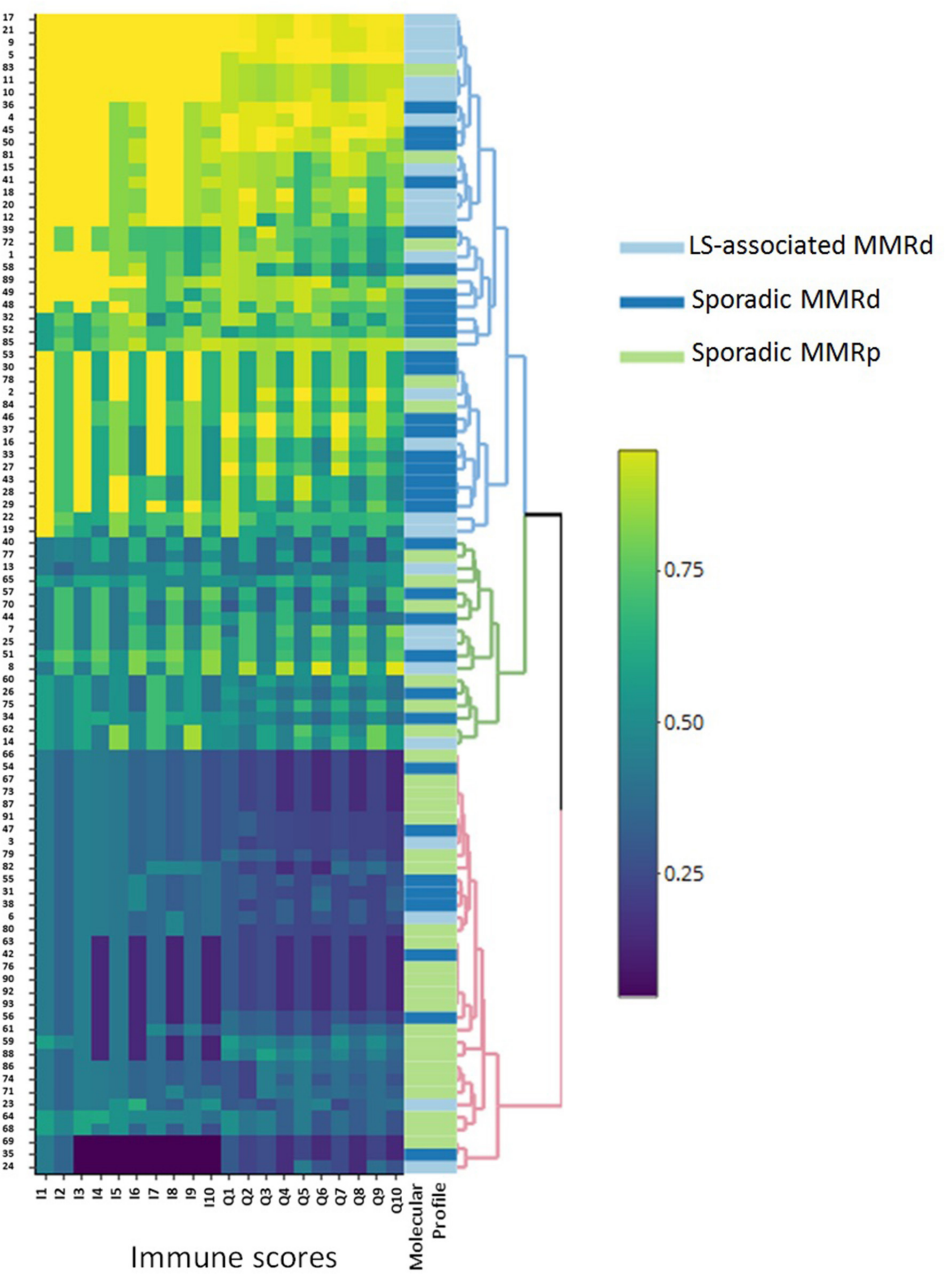
Heatmap outlining the clustering of the molecular groups by immune score. This figure clearly illustrates the broad immune profile of sporadic MMRd loss ECs, as they are seen to infiltrate around both MMRp and LS-associated MMRd groups. LS-associated MMRd, Lynch Syndrome-associated mismatch repair deficient; Sporadic MMRd, Sporadic mismatch repair deficient; MMRp, mismatch repair proficient.

Of note Galon's “Immunoscore” was not significantly different between the LS-associated MMR-deficient vs. sporadic MMR-deficient cohorts. However, Q2, Q4, Q6, Q8, and Q10 were significantly different across the three cohorts (Table 4 and Figure 5). These scores informed machine learning-based predictive models.

**Table 4:** Immune score performance in distinguishing the three molecular cohorts in both two and three class analysis.

	**Dunn's Test Kruskal-Wallis & BH (3-class)**			**Kruskal-Wallis (2-class)**
**Score**	**LS-associated vs. sporadic MMRd**	**LS-associated vs. MMRp**	**Sporadic MMRd vs. MMRp**	**MMR Deficient vs. MMR Proficient**
I1	0.2884	**0.0031**	**0.005**	**0.00075**
I2	0.0738	**0.0001**	**0.005**	**0.00012**
I3	0.4213	**0.0045**	**0.009**	**0.00129**
I4	0.1115	**0.0003**	**0.0049**	**0.00019**
I5	0.1857	**0.0021**	**0.0099**	**0.00102**
I6	0.0583	**0.0001**	**0.0044**	**0.00007**
I7	0.1226	**0.0005**	**0.0072**	**0.00038**
I8	**0.0248**	**<0.0001**	**0.0087**	**0.00009**
I9	0.0531	**0.0002**	**0.0135**	**0.0004**
I10	**0.0189**	**<0.0001**	**0.0062**	**0.00004**
Q1	0.3072	**0.0008**	**0.001**	**0.0001**
Q2	**0.0461**	**<0.0001**	**0.0017**	**0.00001**
Q3	0.3205	**0.0054**	**0.0063**	**0.00121**
Q4	0.369	**0**	**0.0071**	**0.0001**
Q5	0.1414	**0.0014**	**0.0127**	**0.00103**
Q6	**0.0202**	**<0.0001**	**0.009**	**0.00008**
Q7	0.1461	**0.0012**	**0.0105**	**0.00081**
Q8	**0.0155**	**<0.0001**	**0.0095**	**0.00007**
Q9	0.0629	**0.004**	**0.0186**	**0.00078**
Q10	**0.0087**	**<0.0001**	**0.0111**	**0.00006**

*LS-associated MMRd, Lynch Syndrome-associated mismatch repair deficient; Sporadic MMRd, Sporadic mismatch repair deficient; MMRp, mismatch repair proficient*.

*All statistically significant results (p < 0.05) are in bold*.

### Predictive Modeling and Classification

Three-class predictive modeling (LS-associated MMR-deficient vs. sporadic MMR-deficient vs. MMR-proficient) presented challenges due to aforementioned masking of LS-associated and MMR-proficient phenotypes by the sporadic MMR-deficient profile (Figure 5). Standard binary immune scores (I1:I10) were unable to classify any three-class groupings by machine learning methods. Our high-resolution quaternary score Q2 was the only immune score to offer three-class predictive potential; however this was viable only in neural network models and still did not approach accuracies above 0.69 by any train control or cross-validation process. Multinomial logistic regression, discriminant analysis, and support vector machines were all unsuccessful in mitigating masking the sporadic MMR-deficient group presented.

**Figure 6:**
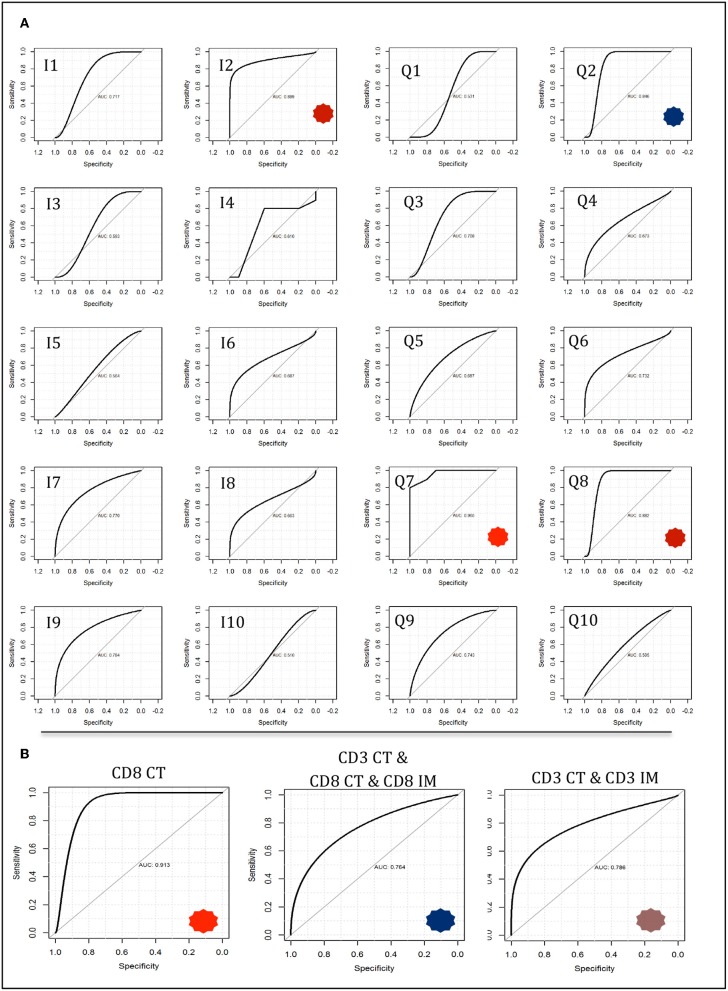
Neural network modeling for two-class response variables of mismatch repair deficient vs. mismatch repair proficient. Models were trained on a 50 observation random subset and tested on a 20 observation validation data subset as described in methods and described further in Supplementary Appendixes 6–9. **(A)** Immune score predictive accuracy from the tumor core compartment only generated from neural network modeling. Receiver operating characteristic (ROC) curves are shown with notable AUCs of 0.899 (I1), 0.846 (Q2), 0.965 (Q7), and 0.882 (Q8) depicted with asterisks. **(B)** Neural network models constructed using feature counts instead of immune scores. Select ROC curves are shown with AUCs of 0.913 (CD8 CT); 0.786 (CD3 CT, CD3 IM); 0.784 (CD3 CT, CD8 CT, CD8 IM).

By contrast, when CD3^CT^, CD8^CT^, and CD8^IM^ were used as explanatory features for two-class prediction potential (MMR-deficient vs. MMR-proficient) neural network and linear support vector machine models achieved high accuracies. Neural network modeling with either Q7 or Q8 high-resolution immune scores provided accuracies of 0.85, wherein immune score Q7 presented an AUC of 0.965 (sensitivity 70%, specificity 100%, PPV 1, NPP 0.77) (Figure 6A). The equivalent binary I7 provided an AUC of 0.770 (sensitivity 70%, specificity 80%, PPV 0.78, NPP 0.27). Fisher Discriminant Analysis provided accuracies concordant with those derived by neural network modeling (0.85) when Q7 or Q8 immune scores were used as explanatory features.

Of note, a 0.8 accuracy was consistently achieved in two-class analysis (MMR-deficient vs. MMR-proficient) with the use of only CD8^CT^ T-cell count by three different classification methods: Feed forward Neural Networks (AUC 0.913); Linear Support Vector Machines (AUC 0.912); and Fisher Discriminant Analysis (AUC 0.8) with observed concordant metrics (sensitivity 70%, specificity 90% PPV 0.88 NNP 0.75). Figure 6B depicts neural network AUCs for the three most suitable raw count selections. Indeed, CD8^CT^ performed best across all modeling strategies, whilst inclusion of any additional immunological markers correlated with decreased accuracy (detailed in Supplementary Appendixes 6–9).

## Discussion

In this study, we found highly significant differences in immune cell infiltrates in endometrial tumors according to their MMR status and origin. Most strikingly, LS-associated MMR-deficient EC had significantly more PD-1+, CD8+, and CD45RO+ infiltrating immune cells at the IM than sporadic MMR-deficient tumors. Furthermore, machine learning algorithms were able to discriminate within and between MMR-deficient and proficient tumors with strong predictive accuracy when high resolution immune scores were used. Of particular note, tumor center CD8+ T-cell counts alone were sufficient to reliably predict the MMR status and origin of endometrial tumors using these models.

Taken together, these data support the notion that whilst MMR deficiency is an important predictor of local immune response in EC, the distinction between an inherited and sporadic cause of MMR deficiency is critical. Both inherited and sporadic MMR deficiency lead to an abundance of FSPs and tumor specific neoantigens, which are thought to drive anti-cancer immunogenicity (37). Whilst sporadic tumors arise without chronicity, however, pathogenic MMR carriers develop clones of MMR-deficient tumor cells that regress spontaneously throughout life (38). This phenomenon likely reflects the successful intervention of primed immune effectors that eliminate tumor clones presenting FSP-epitopes on their cell surface (39). Consequently, a LS-associated tumor must develop sophisticated immune evasion mechanisms to avoid natural clearance, to allow them to grow and present clinically (24). Our data support this concept since we observed a significantly higher density of infiltrating immune cell effectors in LS-associated compared with sporadic MMR-deficient EC. Furthermore, LS-associated tumors showed significantly higher counts of CD45RO+ T-cells, indicating the presence of sustained anti-tumor immune response mechanisms at play. A further key finding was the significantly higher number of PD-1+ T-cells in LS-associated compared to sporadic MMR-deficient endometrial tumors. This supports the “chronicity” argument of LS-associated MMR-deficient EC, and is of clinical significance given the emergence of PD-1 blockade-based chemotherapeutic agents, for example pembrolizumab.

Two seminal reports found immune checkpoint inhibitors are more potent against MMR-deficient tumors (28, 29). LS carriers were disproportionately represented in both trials, with as many as 48% of participants having LS in one of the studies (29). By contrast, the proportion of LS in CRC (40) and EC populations (41, 42) is around 3%. Our data would support the theory that LS-associated MMR-deficient tumors are more sensitive to immune checkpoint blockade. Thus, it may be prudent to explore PD-1 blockade in the context of germline vs. sporadic MMR-deficient tumors before extrapolating the results of these trials to the general cancer population.

Differences in infiltrating immune cells between the tumor groups underpinned the rationale for our machine learning modeling. In our study, Galon's “Immunoscore” did not distinguish LS-associated from sporadic MMR-deficient tumors despite significant differences in individual immune effector populations between the two. By contrast, higher resolution Q-scores were able to reliably distinguish between LS-associated and sporadic MMR-deficient tumors. We noted considerable immunological heterogeneity between the tumors of the sporadic MMR-deficient group; their immune cell counts variously clustered around the LS-associated MMR-deficient and MMR-proficient tumors (Supplementary Appendix 8, Figure S14). Predictive modeling was unable to resolve such variance into three-class *LS-associated vs. sporadic MMR-deficient vs. MMR-proficient tumors* classification with clinically-relevant discriminatory power. However, neural network modeling was able to accurately predict a tumor's MMR status and origin based purely on CD8+ tumor core count (CD8^CT^), a finding with potential for clinical application. As the “Immunoscore” becomes routinely measured to guide treatment decisions, entry into clinical trials and individualized prognostic information (14, 20, 21, 43), a CD8^CT^ threshold density could trigger reflex MMR testing from a diagnostic biopsy specimen. With the use of automated IHC and automated scoring software, this process could be almost entirely automated.

Our work builds on the work by Pakish et al., who also call for LS-associated and sporadic MMR-deficient tumors to be considered separate entities in future clinical trials (9). They too noted higher CD8+ immune cells in presumed LS-associated compared with sporadic MMR-deficient endometrial tumors, however they were not able to confirm LS status through germline sequencing. Two somatic mutations in one of the MMR genes, or “loss of heterozygosity,” is observed twice as often as germline mutations in MMR-deficient tumors that do not show *MLH1* hypermethylation (44). As somatic mutations are an acute event, they may not prime the immune response as expected in LS. In contrast to our findings, Pakish et al. found reduced PD-L1 expression in presumed LS-associated MMR-deficient tumors, but did not measure PD-1+ T-cell density, which complicates interpretation of their findings. Furthermore, by failing to identify important tumor compartments when choosing their ROI, it was not possible for Pakish et al. to decipher intra-tumoral differences that have been shown to have prognostic implications in other tumor sites (21). Thus, we chose ROI by identifying regions of the core and invasive margin of the tumor with the highest CD3+ T-cell densities and used sequential sections of the same ROI for all immune cell markers, to ensure geographically homogeneity. This allowed us to capture intra-tumoral immune cell interactions, without the need for multiplex IHC.

Strengths of our study include the large cohort of tumors from women with known LS. All LS-associated tumors showed loss of the expected MMR protein(s) on IHC that corresponded to their germline pathogenic variant. Using the LS tumors as cases, we were able to match control tumors to important pathological variables that in themselves could influence immune response, thus reducing confounding. Immune effectors were double scored and manually counted and sophisticated machine learning algorithms were able to identify CD8+ tumor core count as a reliable predictor of MMR status and origin, which could have clinical utility.

Weaknesses of this study include inclusion of only one tumor from a *PMS2* pathogenic variant carrier, who had constitutional mismatch repair deficiency (32). *PMS2*-associated EC and CMMRD are rare clinical entities (31, 45, 46), and thus we would recommend caution in the extrapolation of our data to these populations. The histological subtypes of tumors varied across the cohorts, however, restricting analysis to the endometrioid tumors only did not change the results. We did not undertake any *in vitro* analysis to determine immune cell function, but included CD45RO+ and PD-1+ markers in our IHC panel, which are well-established T-cell activation markers (47, 48). Our two-class machine learning models used a 50-observation training subset and a 20-observation test subset for validation. We attempted to mitigate model inaccuracy and overfitting through cross-validation (and explanatory feature pre-processing and model tuning where relevant), however we acknowledge that our dataset may not represent the wider genomic profile population of mismatch repair pathologies. We are reassured that multiple different models (2,600 models were tested) produced concordant outcomes but accept that our model would benefit from validation with larger datasets. Whilst the LS cohort was significantly younger than the other cohorts, adjusting for age did not influence the results. We were not able to compare survival outcomes in our study due to its small numbers and the inherent bias of comparing archival LS tumors with prospectively collected sporadic tumors.

In conclusion, this is the first study looking at the immune microenvironment in confirmed LS- associated EC. We have shown that LS-associated and sporadic MMR-deficient ECs are distinct immunological entities and therefore should be treated as such in clinical trials. Furthermore, the current evidence for PD-1 checkpoint inhibitors should be re-evaluated with LS-associated and sporadic MMR-deficient EC-specific outcomes reported. Our data suggest that LS-associated EC might have a more favorable outcome than the more common sporadic MMR-deficient EC. Moreover, we have shown the potential of machine learning modeling to explore differences in tumor biology. Through such modeling we have found CD8^CT^ cell count can predict MMR deficiency in endometrial tumors to a level approaching clinical utility. Indeed, with the increasing popularity of Galon's “Immunoscore,” this could enable a novel means of identifying, and risk stratifying MMR-deficient tumors.

## Data Availability Statement

All raw data can be made available on request. Image data requests will take longer to process, given the data file size. All images need to be viewed with the CaseViewer software, which is free to download at: https://www.3dhistech.com/caseviewer.

## Ethics Statement

The study was approved by the North Lancaster Research Ethics Committee (ref: 15/NW/0733 and 16/NW/0164) and sponsored by the University of Manchester, UK. All participants gave written, informed consent for their data and tumor tissue to be used for research.

## Author Contributions

NCR, NAJR, and EC designed the study. NCR, NAJR, TW, LH, JB, and TB acquired, analyzed, and interpreted the data. NAJR and TW performed the statistical analyses. TW performed the machine learning modeling. DE, TB, and EC supervised the study. NCR, NAJR, TW, and EC wrote the manuscript. All authors provided critical comment and approved the final version of the manuscript. NCR, NAJR, DE, and EC obtained funding. EC is the study guarantor.

### Conflict of Interest

The authors declare that the research was conducted in the absence of any commercial or financial relationships that could be construed as a potential conflict of interest.
