# Supplementary material for: Distinct Immunological Landscapes Characterize Inherited and Sporadic Mismatch Repair Deficient Endometrial Cancer
Source: Front Immunol. 2020 Jan 9;10:3023. doi: 10.3389/fimmu.2019.03023 (PMC6970202; doi:10.3389/fimmu.2019.03023)
Supplement: Supplementary file 1 [file Data_Sheet_1.docx]

**Distinct immunological landscapes characterize inherited and sporadic mismatch repair deficient endometrial cancer**

**Supplementary Material**

Table of Contents

Appendix 1: Mismatch Repair Proteins expression, Microsatellite Instability Analysis, and MLH1 Hypermethylation Analysis 3

1.1 Immunohistochemistry (IHC) for mismatch repair proteins 3

1.2 Microsatellite Instability Analysis 5

1.3 MLH1 Hypermethylation Analysis 6

1.3.1 Sequence of MLH1 region of interest 6

1.3.2 PCR detection of MLH1 hypermethylation 6

Appendix 2. Optimization of Antibodies 7

Appendix 3: Optimized immunohistochemistry 8

Figure S1. Optimized CD3 immunohistochemistry protocol and controls. 8

Figure S2. Optimized CD8 immunohistochemistry protocol and controls. 8

Figure S3. Optimized CD45RO immunohistochemistry protocol and controls. 8

Figure S4. Optimized FoxP3 immunohistochemistry protocol and controls. 9

Figure S5. Optimized PD-1 immunohistochemistry protocol and controls. 9

Figure S6. Optimized PD-L1 immunohistochemistry protocol and controls. 9

Appendix 4: Immune cell scoring 10

Figure S7. Identification of scoring compartments. 10

Appendix 5: Q-Q scores showing the distribution of immune marker count scores. 11

Figure S8 Q-Q plots as to explore the data distribution. 15

Appendix 6: Machine learning predictive model assessment: three-class or two-class analysis of Immune scores 16

Figure S9: Schema of machine learning model assessment for use of Immune scores as explanatory features in three-class or two-class predictions. 16

Appendix 7: Machine learning modeling overviews for three-class and two-class analysis of raw counts in selected cohorts 17

Figure S10: Schema of machine learning model assessment for use of raw counts as independent explanatory features in three-class predictions. 17

Figure S11: Schema of machine learning model assessment for use of raw counts as independent explanatory features in two-class predictions 18

Appendix 8: Key outputs from three-class machine modeling of immune scores and scores 19

Figure S13: Heat map of neural network multinomial logistic regression outputs (counts) with associated AUC for CD8+FOXP3+PD1+PDL1 tumor core counts 20

Figure S14: Graph of Discriminant function scores for Lynch Syndrome-associated mismatch repair deficient (LS-associated MMRd), Sporadic MMRd, and Sporadic MMRp stratification. 21

Appendix 9: Machine learning modeling outputs for two-class analysis 22

Figure S15: Tabular outputs of neural network machine learning two-class analysis (mismatch repair deficient (Sporadic MMRd) vs. mismatch repair proficient (Sporadic MMRp) Immune scores). 22

# **Appendix 1:** Mismatch Repair Proteins expression, Microsatellite Instability Analysis, and MLH1 Hypermethylation Analysis

## 1.1 Immunohistochemistry (IHC) for mismatch repair proteins

IHC was performed by the pathology staff at Manchester University Foundation NHS Trust (MFT) on the automated Ventana BenchMark ULTRA IHC ⁄ ISH Staining Module (Ventana Co., Tucson, AZ, USA) within the pathology department at MFT. The automated Ventana BenchMark ULTRA IHC ⁄ ISH Staining Module (Ventana Co., Tucson, AZ, USA) was used together with the OptiView, 3’ diaminobenzidine (DAB) version 5 detection system (Ventana Co.). Tissue sections (4 µm) were deparaffinized and incubated in EZPrep Volume Adjust (Ventana Co.). At intervals between steps the slides were washed with a TRIS-based Reaction Buffer, pH 7.6. A heat-induced antigen retrieval protocol set for 64 min was carried out using a TRIS– ethylenediamine tetracetic acid (EDTA)–boric acid pH 8.4 buffer (Cell Conditioner 1). The sections were incubated with OptiView Peroxidase Inhibitor for 4 min, then with antibody to PMS2 for a set time of 60 min at room temperature. This was followed by incubation with OptiView HQ Universal Linker secondary antibody (8 min.), followed by OptiView HRP Multimer tertiary anti-HQ antibody HPR-labelled for 8 min, then DAB chromogen and substrate (8 min.), and copper enhancer for 4 min. Counterstain (haematoxylin II) was applied for 12 min before an incubation of 4 min with bluing reagent. Slides were then dehydrated through 99% IDA and Xylene and coverslipped with a xylene based mountant

MLH-1 Protocol Details

Antibody: Anti-MLH-1 (M1) Mouse Monoclonal

Manufacturer: Ventana

System: Automated Ventana Benchmark ULTRA

Detection Kit: OptiView DAB IHC Detection System

Antigen Retrieval: 24 minute heat in CC1 (Tris based pH8.4 buffer)

Antibody concentration: RTU (Ready to use)

Antibody Incubation: 16 minute at room temperature.

Counterstain: Haematoxylin 12 min, Bluing Reagent 4 min.

Positive Tissue Control: Colon

MSH2 Protocol Details

Antibody: MSH2 (G219-1129) Mouse Monoclonal

Manufacturer: Cell Marque

System: Automated Ventana Benchmark ULTRA

Detection Kit: OptiView DAB IHC Detection System

Antigen Retrieval: 32 minute heat in CC1 (Tris based pH8.4 buffer)

Antibody concentration: 1+50

Antibody Incubation: 16 minute at room temperature.

Counterstain: Haematoxylin 12 min, Bluing Reagent 4 min.

Positive Tissue Control: Colon

MSH6 Protocol Details

Antibody: MSH6 (SP93) Rabbit Monoclonal

Manufacturer: Cell Marque

System: Automated Ventana Benchmark ULTRA

Detection Kit: OptiView DAB IHC Detection System

Antigen Retrieval: 32 minute heat in CC1 (Tris based pH8.4 buffer)

Antibody concentration: 1+50

Antibody Incubation: 16 minute at room temperature.

Counterstain: Haematoxylin 12 min, Bluing Reagent 4 min.

Positive Tissue Control: Colon

PMS2 Protocol Details

Antibody:                               Anti-PMS2

Manufacturer:                          Ventana (760-4531)

System:                                   Automated Ventana Benchmark ULTRA

Detection Kit:                         OptiView DAB IHC Detection System

Antigen Retrieval:                 64 minute heat in CC1 (Tris based pH8.4 buffer)

Antibody concentration:       RTU (Ready to use)

Antibody Incubation:               32 minute at room temperature

Additional Kit: Optiview Amplification 4 min, Optiview Amplification Multimer 4 Min

Counterstain:                         Haematoxylin II 12 min, Bluing Reagent 4 min.

Positive Tissue Control:         Colon

## 1.2 Microsatellite Instability Analysis


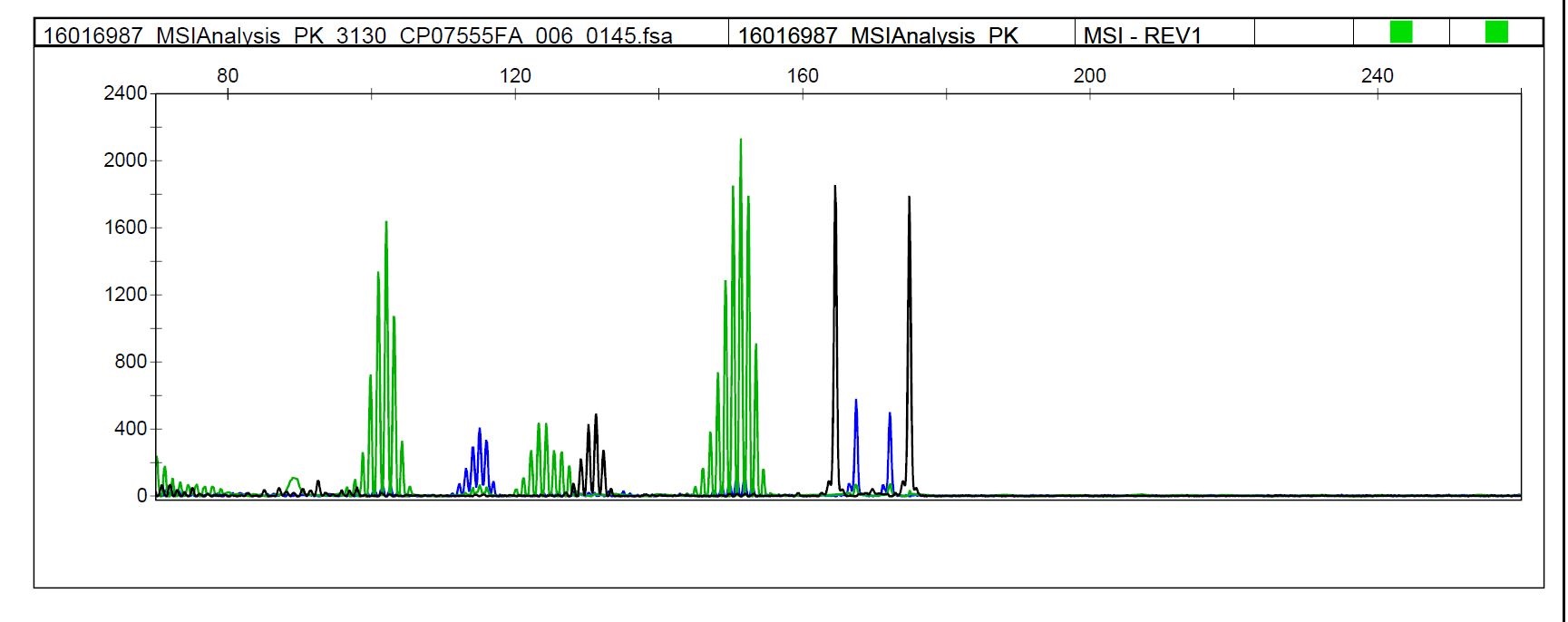


The Microsatellite Instability (MSI) Analysis system.

Graphical representation of capillary electrophoresis of PCR amplified repeat sequences using the MSI Analysis system

**x-axis**: base size; **y-axis**: fluorescent intensity. **A:** Microsatellite instability low (MSI-L). **B:** Microsatellite instability high (MSI-H). Following polymerase chain reaction (PCR) amplification at 5 mononucleotide repeat loci (NR-21, BAT-26, BAT-25, NR-24, MONO-27) and two pentanucleotide repeat loci (Penta C and Penta D), the samples are analysed by capillary electrophoresis. Differences in the x-axis location of fluorescent signals at the corresponding mononucleotide loci in tumor DNA relative to normal DNA indicate sequence expansion or contraction caused by the insertion or deletion of nucleotides, respectively. In **B**, there are contractions at all the mononucleotide repeat loci, classing this tumor as MSI high (MSI-H).

A

##
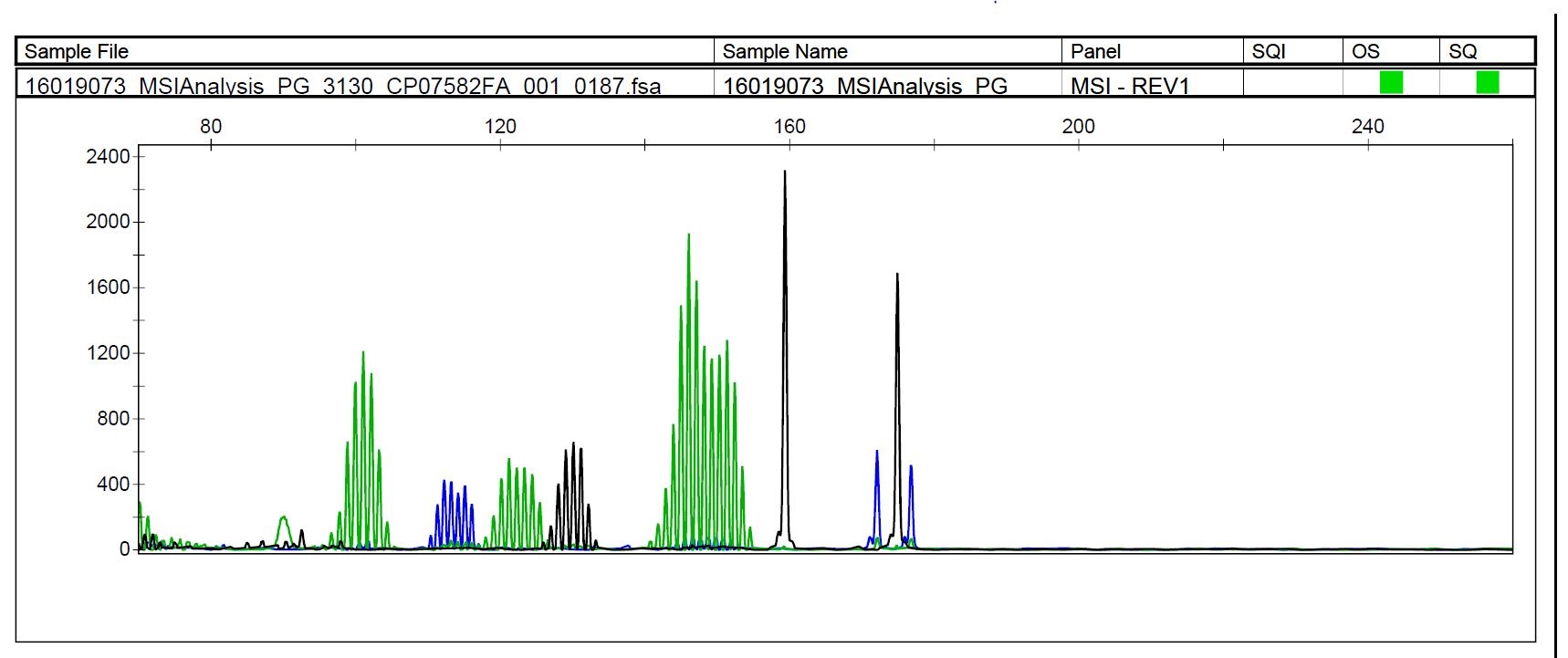


B

## 1.3 MLH1 Hypermethylation Analysis

### 1.3.1 Sequence of MLH1 region of interest

MLH1 Primers region of interest -248 to -178 from Deng *et al* 19993 {Deng:1999wc}. Seq. ref U83845 not same as latest NM_000249.3

CGTCGATTTTTATTTTGTTTTTTTTGGGCGTTATTTATATTTTGCGGGAGGTTATAAGAGTAGGGTTAACGTTAGAAAGGTCGTAAGGGGAGAGGAGGAGTTTGAGAAGCGTTAAGTATTTTTTTCGTTTTGCGTTAGATTATTTTAGTAGAGGTATATAAGTTCGGTTTCGGTATTTTTGTTTTTATTGGTTGGATATTTC

- 248 GTATTTTTCGAGTTTTTAAAAACGAATTAATAGGAAGAG**T/CG**GA**T/C**AG**T/CG**ATTTTTAA**T/CGT/CG**TAAGCGTATA -178 TTTTTTTAGGTAGCGGGTAGTAGTCGTTTTAGGGAGGGACGAAGAGATTTAGTAATTTATAGAGTTGAGAAATTTGATTGGTATTTAAGTTGTTTAATTAATAGTTGTCGTTGAAGGGTGGGGTTGGATGGCGTAAGTTATAGTTGAAGGAAGAACGTGAGTACGAGGTATTGAGGTG -1

ATTGGTTGAAGGTATTTTCGTTGAGTATTTAGACGTTTTTTTGGTTTTTTTGGCGTTAAA**ATG**TCGTTCGTGGTAGGGGTTATTCGGCGGTTGGACGAGATAGTGGTGAATCGTATCGCGGCGGGGGAAGTTATTTAGCGGTTAGTTAATGTTATTAAAGAGATGATTGAGAATTGGTACGGAGGGAGTCGAGTCGGGTT

Primary MLH1 F and MLH1 Prom R Ext primers

Secondary MLH1 F & R primers

Sequencing primers

Bisulphite conversion nucleotide

Bisulphite conversion control nucleotide

### 1.3.2 PCR detection of MLH1 hypermethylation

Hypermethylation PCR analysis output showing increased methylation signal at the CpG *MLH1* island (96%).


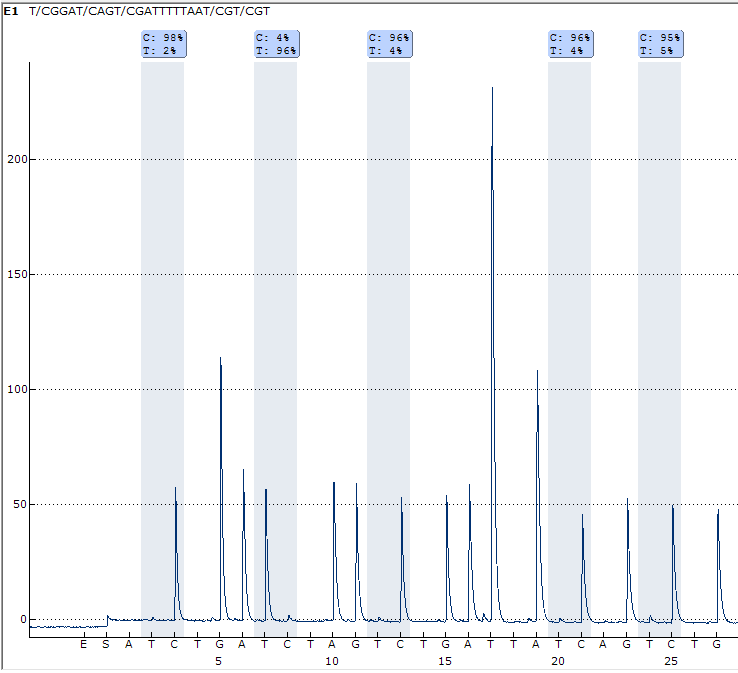


# Appendix 2. Optimization of Antibodies

| **Antibody** | **Clone** | **Company** | **Monoclonal Antibody Species** | **Casein block incubation time** | **Antibody Dilution** | **Antibody Incubation time** | **Isotope Control** | **Positive control tissue** |
| --- | --- | --- | --- | --- | --- | --- | --- | --- |
| CD3  (138mg/L)* | F7.2.38 | DAKO Agilent (California, United States) | Monoclonal mouse anti-human | 30 min | 1:50 | 60 min | Mouse IgG1κ (DAKO Agilent) | Tonsil |
| CD8  (157 mg/L)* | C8/144B | DAKO Agilent | Monoclonal mouse anti-human | 30 min | 1:200 | 60 min | Mouse IgG1κ (DAKO Agilent) | Tonsil |
| CD45RO  (226 mg/L)* | UCLH1 | DAKO Agilent | Monoclonal mouse anti-human | 60 min | 1:200 | 30 min | Mouse IgG2a (DAKO Agilent) | Tonsil |
| PD-1  (1g/L)* | NAT105 | abcam (Cambridge, UK) | Monoclonal mouse anti-human | 30 min | 1:50 | 60 min | Mouse IgG1κ (DAKO Agilent) | Tonsil |
| Fox-P3  (1g/L)* | 236A/E7 | abcam | Monoclonal mouse anti-human | 60 min | 1:100 | 30 min | Mouse IgG1κ (DAKO Agilent) | Tonsil |
| PD-L1  (0.8739 g/L)* | E1L3N | Cell Signalling (Leiden, The Netherlands) | Monoclonal rabbit anti-human | 30 min | 1:400 | 60 min | Rabbit IgGXP  (Cell Signalling) | Placenta |

Optimized immunohistochemistry protocols for use on the automated Bond Max (Leica Biosystems, Wetzlar, Germany) and Bond Rx platform. *Concentration upon delivery.

# Appendix 3: Optimized immunohistochemistry


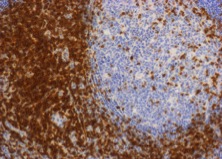


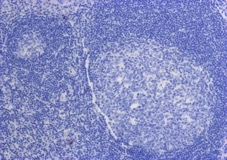

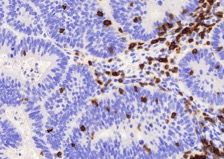


## Figure S1. Optimized CD3 immunohistochemistry protocol and controls.

Left: optimized CD3 protocol (endometrial carcinoma, digitalized image, magnification x20); middle: positive control (tonsil, light microscope image, magnification x10); right: mouse IgG1κ isotype control (tonsil, light microscope image, magnification x10). Arrow points to CD3+ T-cell.


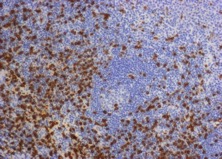

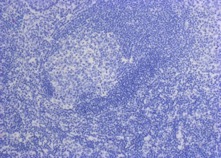

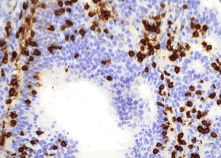


## Figure S2. Optimized CD8 immunohistochemistry protocol and controls.

Left: optimized CD8 protocol (endometrial carcinoma, digitalized image, magnification x20); middle: positive control (tonsil, light microscope image, magnification x10); right: mouse IgG1κ isotype control (tonsil, light microscope image, magnification x10). Arrow points to CD8+ T-cell.


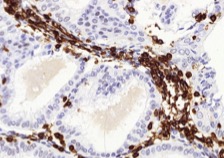

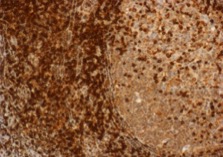

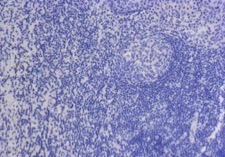


## Figure S3. Optimized CD45RO immunohistochemistry protocol and controls.

Left: optimized CD45RO protocol (endometrial carcinoma, digitalized image, magnification x20); middle: positive control (tonsil, light microscope image, magnification x10); right: mouse IgG2a isotype control (tonsil, light microscope image, magnification x10). Arrow points to CD45RO+ T-cell.

#####
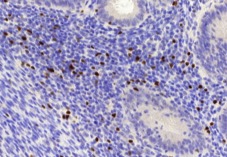

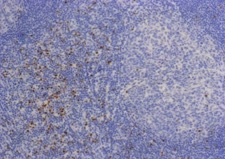

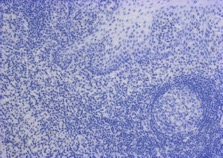


#####

## Figure S4. Optimized FoxP3 immunohistochemistry protocol and controls.

Left: optimized FoxP3 protocol (endometrial carcinoma, digitalized image, magnification x20); middle: positive control (tonsil, light microscope image, magnification x10); right: mouse IgG1κ isotype control (tonsil, light microscope image, magnification x10). Arrow points to FoxP3+ T-cell.


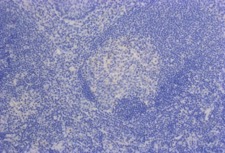

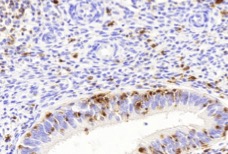

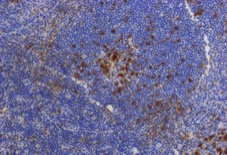


## Figure S5. Optimized PD-1 immunohistochemistry protocol and controls.

Left: optimized PD-1 protocol (endometrial carcinoma, digitalized image, magnification x20); middle: positive control (tonsil, light microscope image, magnification x10); right: mouse IgG1κ isotype control (tonsil, light microscope image, magnification x10). Arrow points to PD-1+ T-cell.


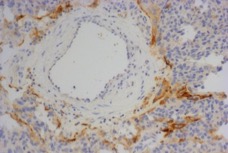

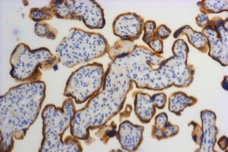

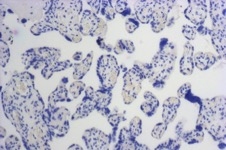


## Figure S6. Optimized PD-L1 immunohistochemistry protocol and controls.

Left: optimized PD-L1 protocol (endometrial carcinoma, light microscope image, magnification x20); middle: positive control (placenta, light microscope image, magnification x10); right: rabbit IgGXP isotype control (placenta, light microscope image, magnification x10). Arrow points to PD-L1+ cell.

# Appendix 4: Immune cell scoring


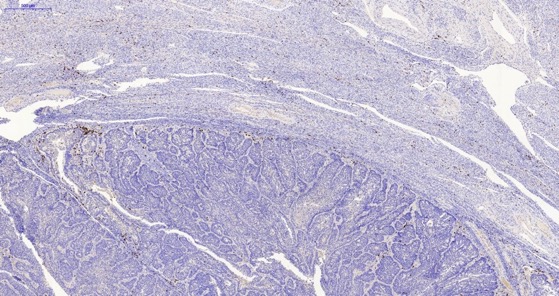


Healthy Tissue (myometrium)

Tumour center (CT)

Invasive margin (IM)

## Figure S7. Identification of scoring compartments.

Regions of interest were selected at the invasive margin and tumor center. Shown here is an example of a digitalized slide stained for CD8, to demonstrate the invasive margin and tumor center. Magnification x4.


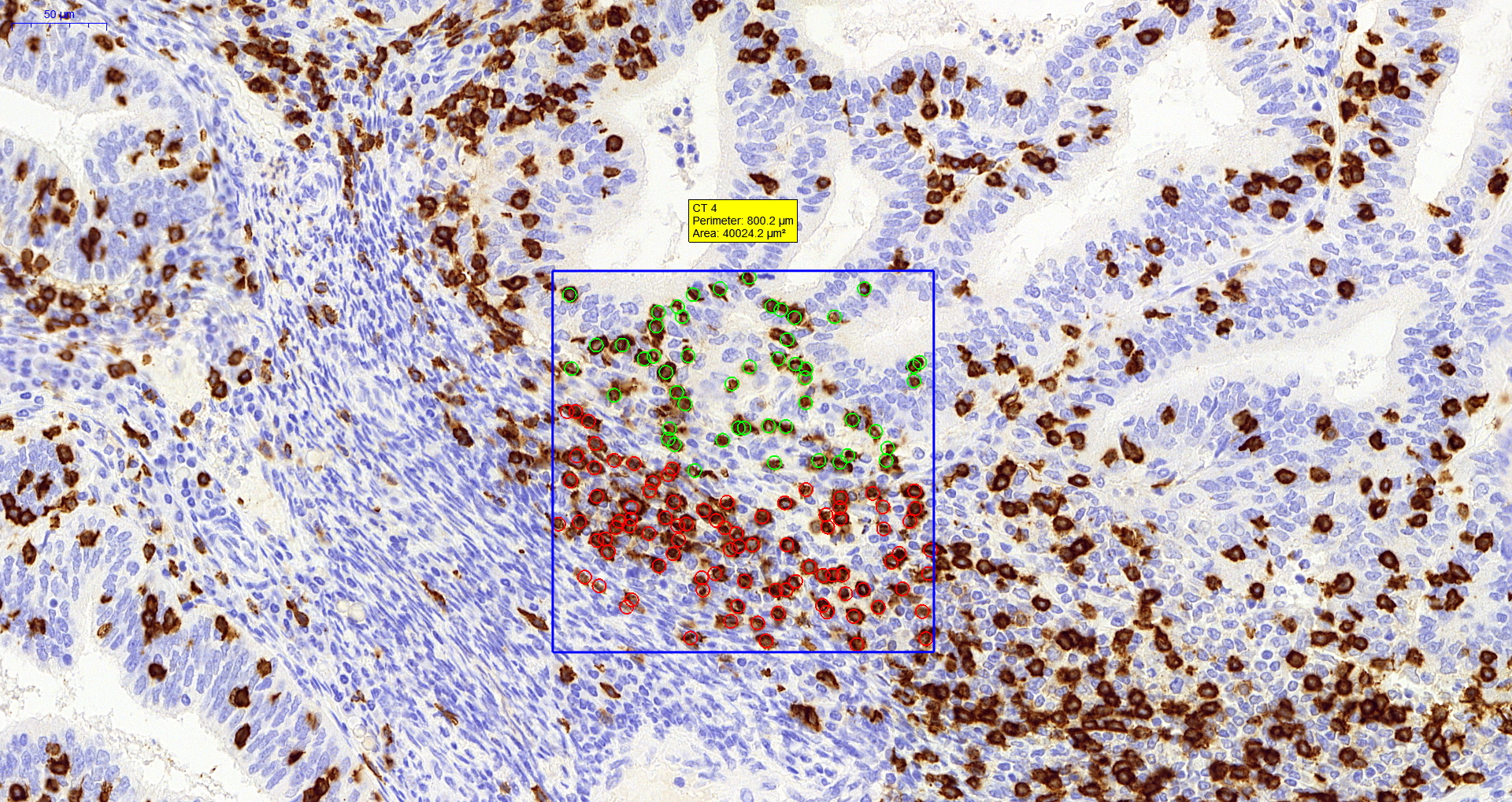


Figure S7b Following slide digitalization, immune-cell markers (CD3, CD8, PD-1 and FoxP3) were scored using the cell counter feature on CaseViewer (3D Histech) as shown here for CD3. For CD3 and CD8, intra-epithelial cells (green) and intra-stromal cells (red) were individually counted at the CT. Magnification x40. CT: tumor center

# Appendix 5: Q-Q scores showing the distribution of immune marker count scores.


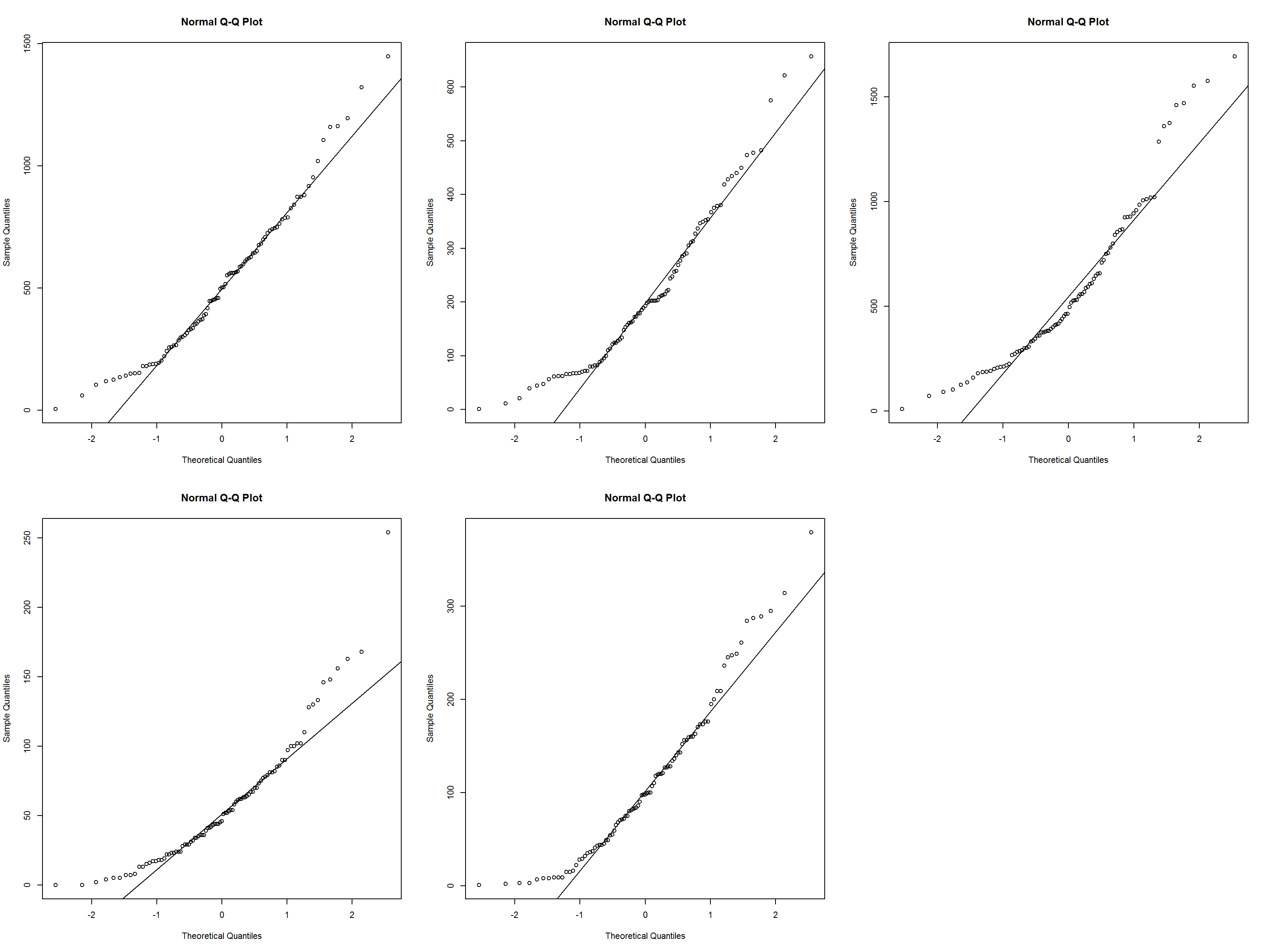


A


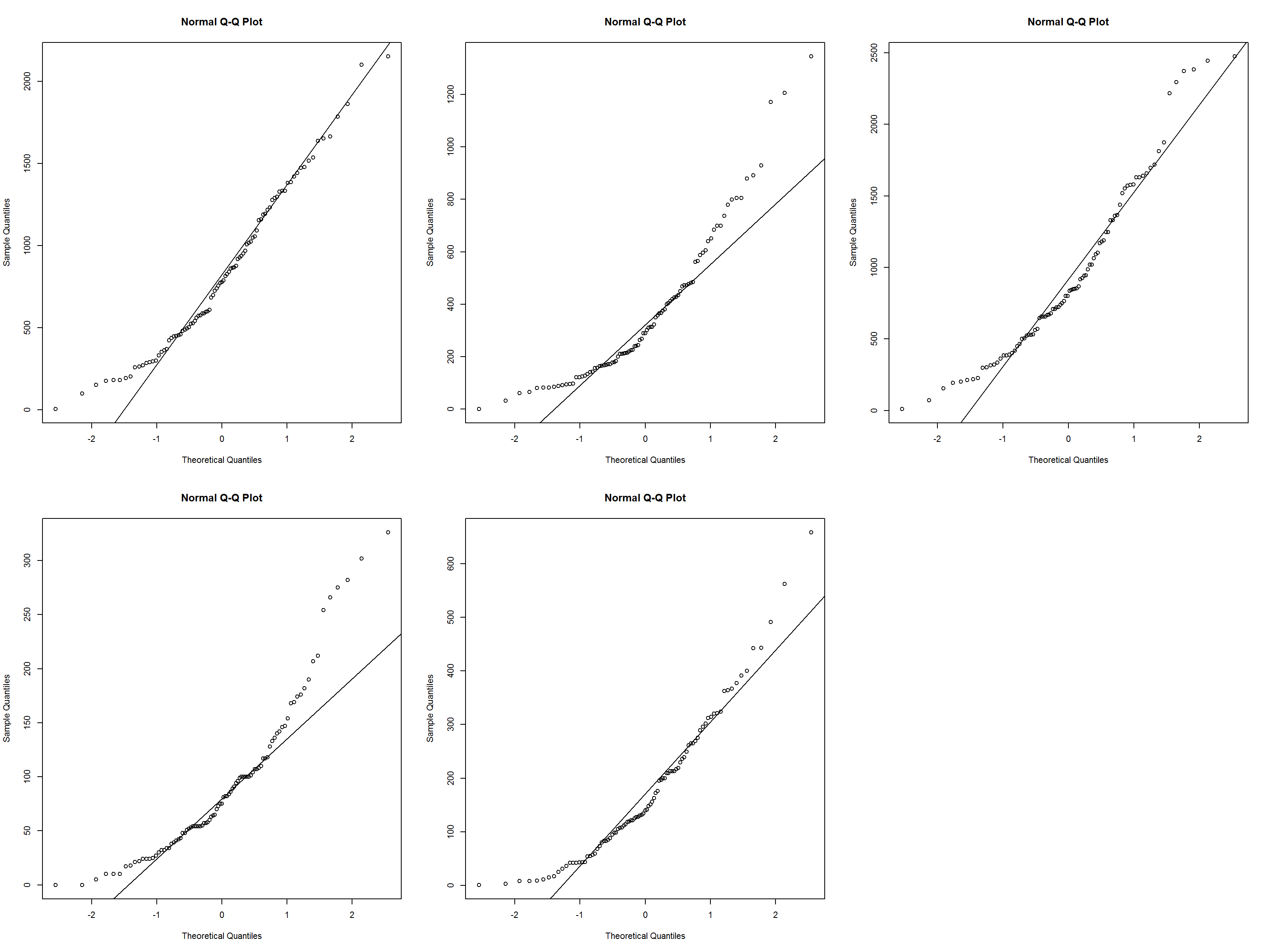


B


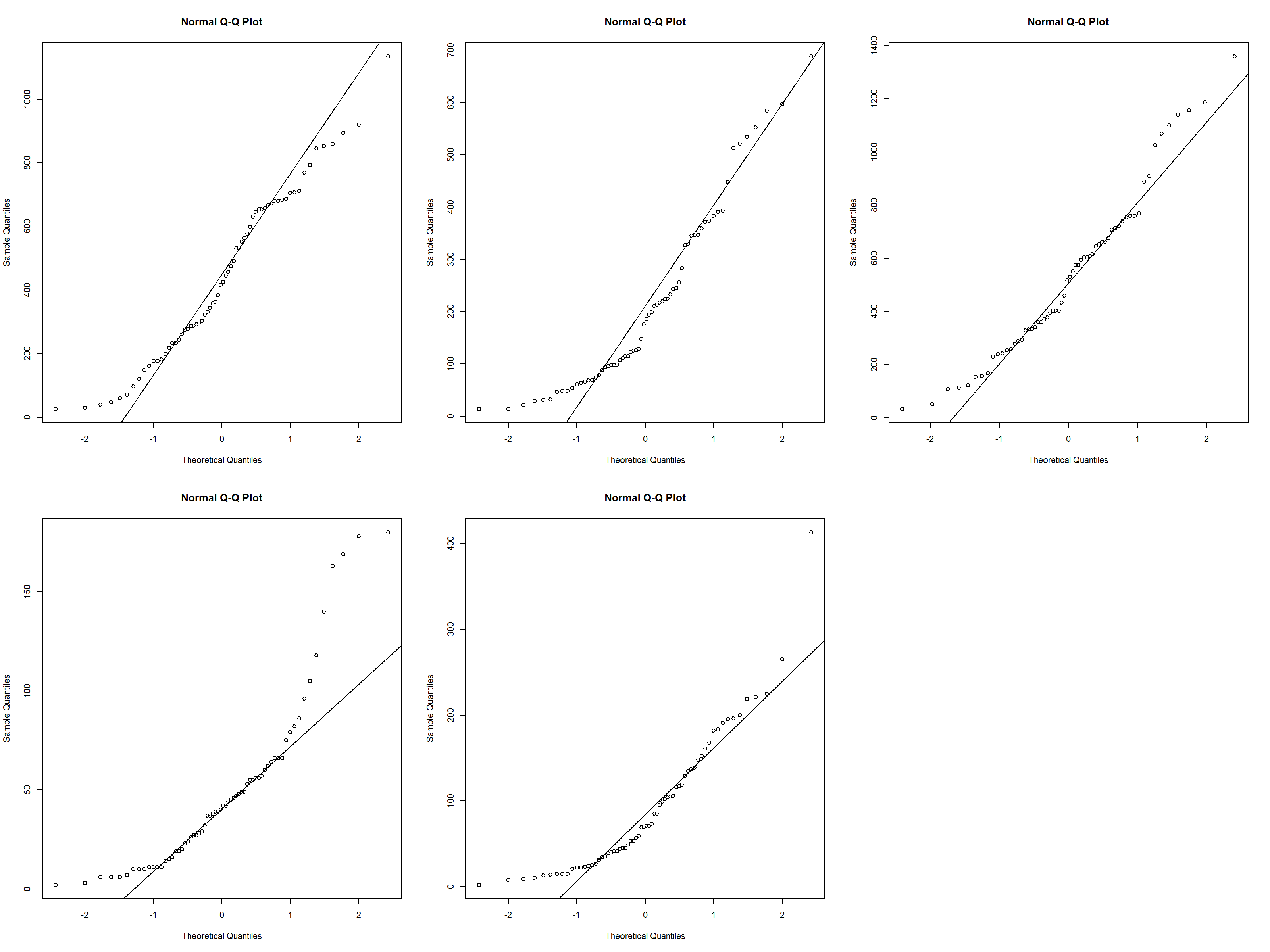


C

D


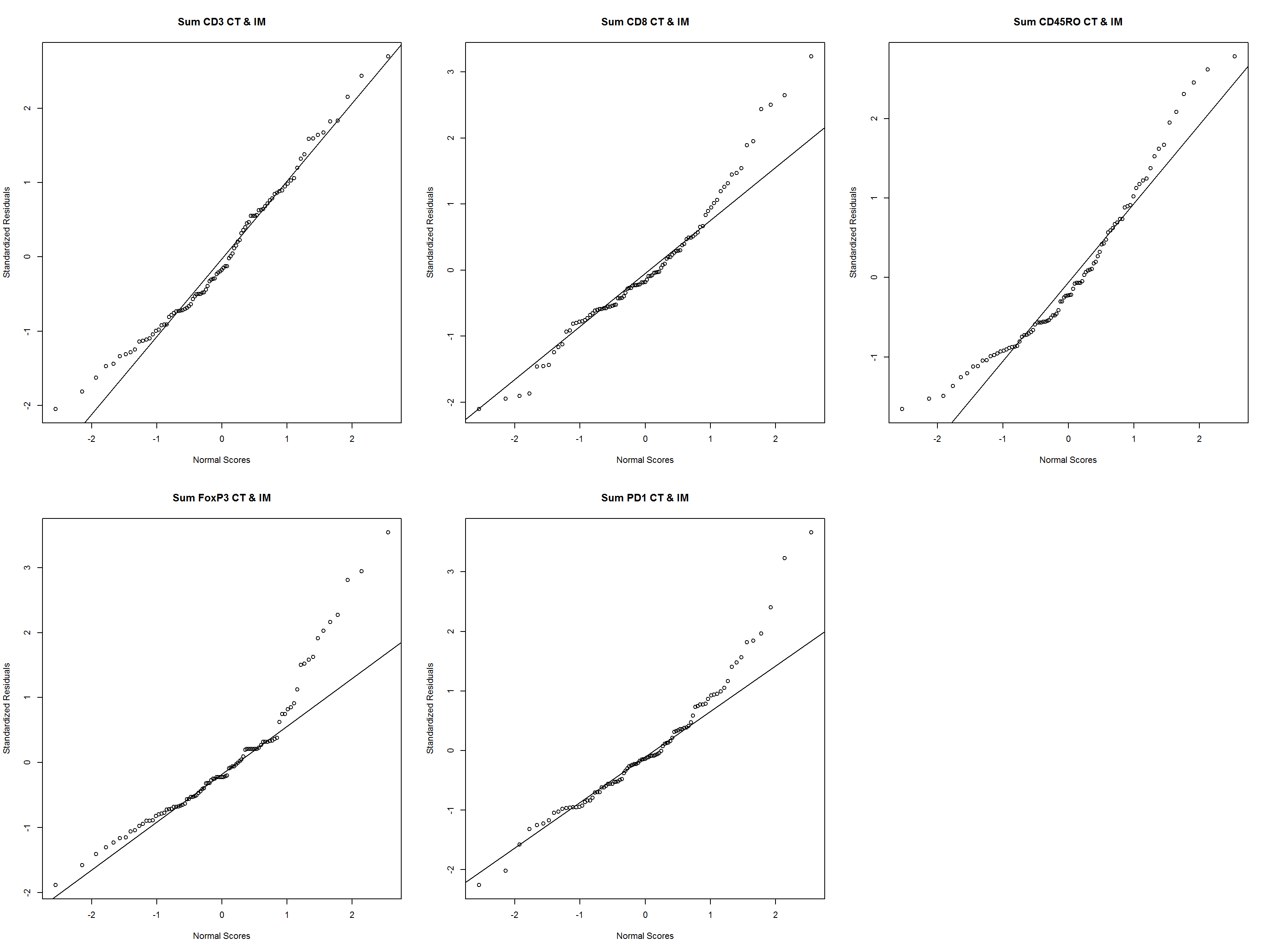


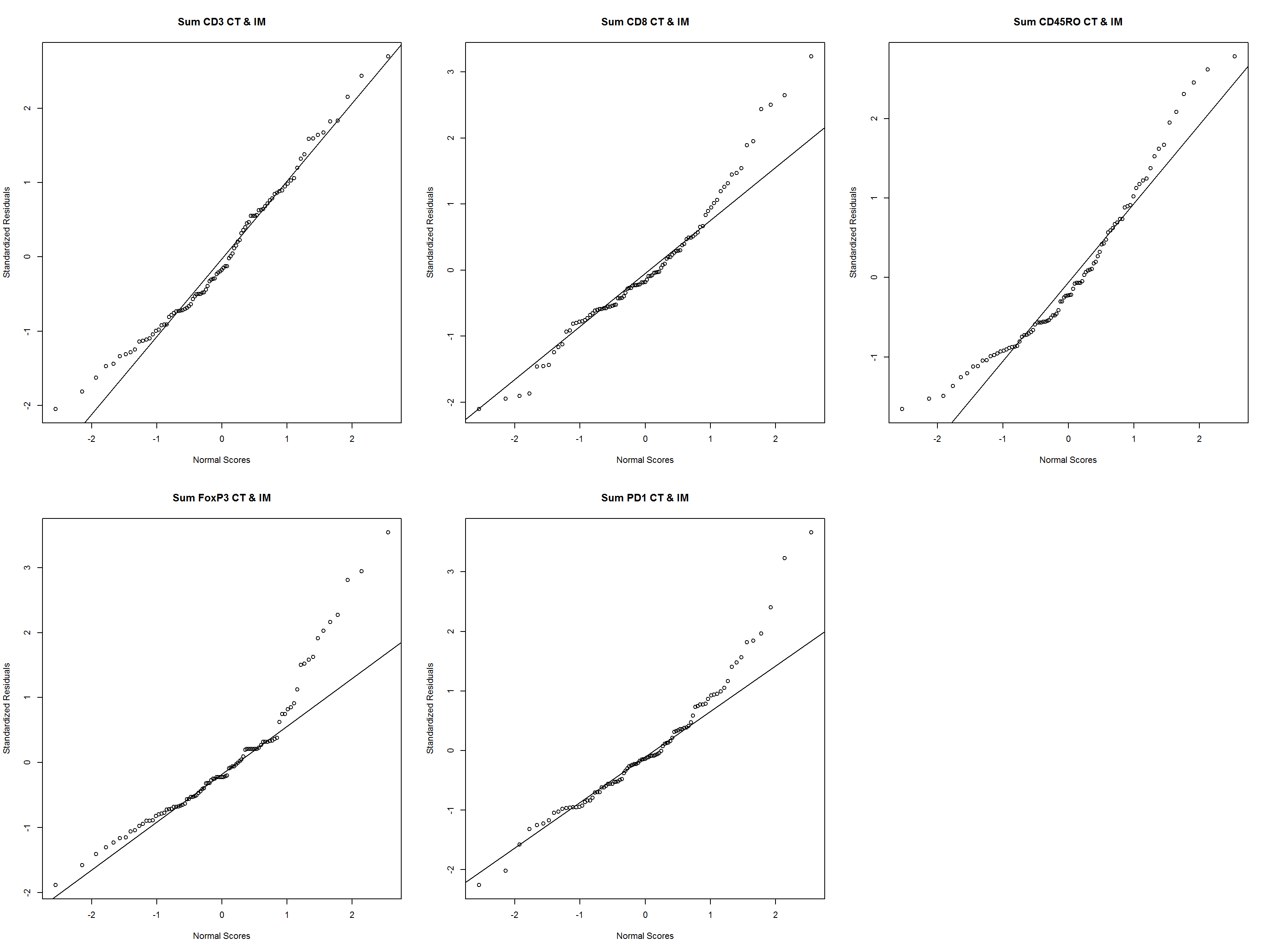


E

F


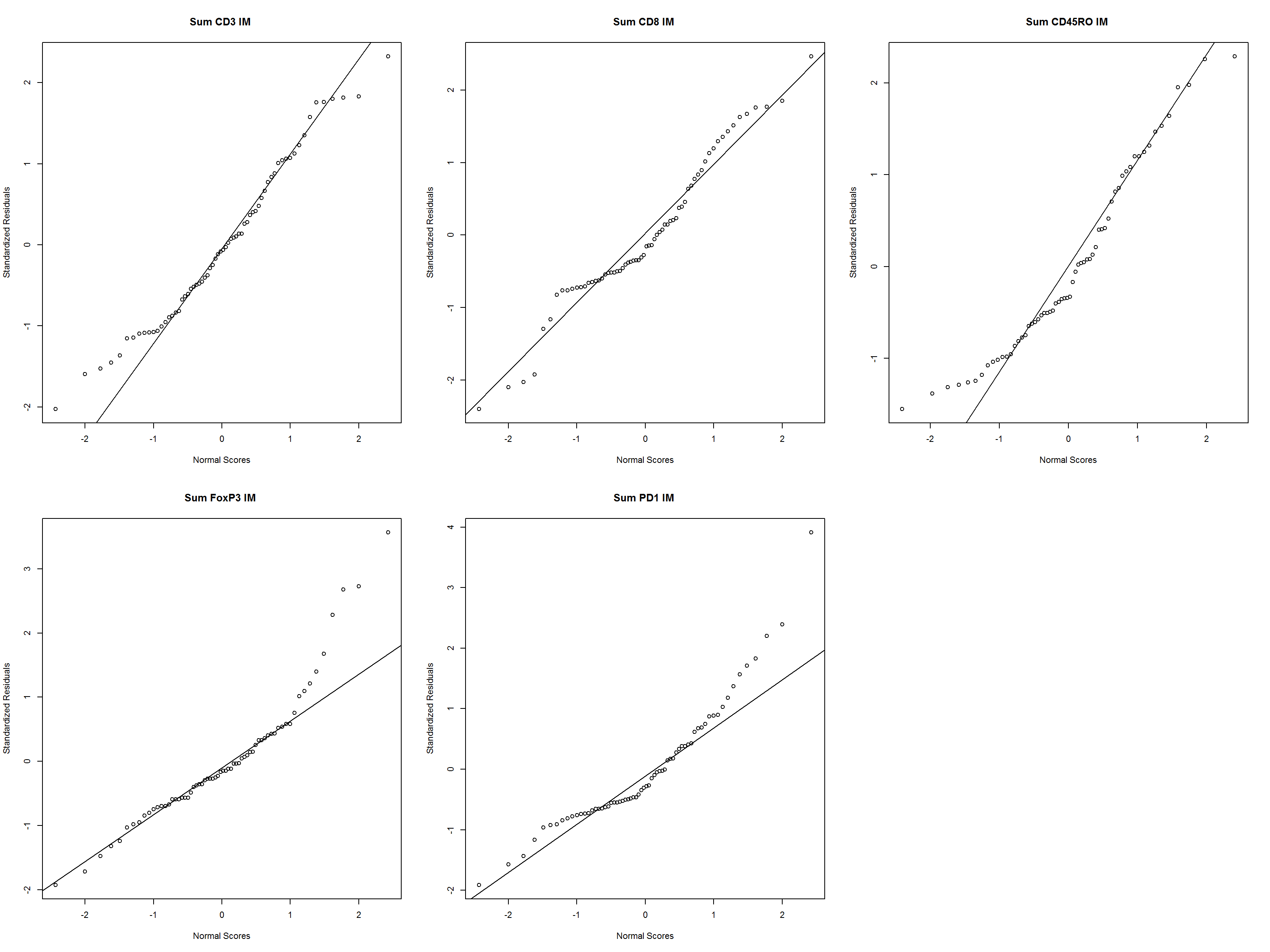


## Figure S8 Q-Q plots as to explore the data distribution.

All data was non-parametrically distributed and therefore non-parametric tests were reported over parametric statistics. A: Q-Q plots for tumor core scores. B: Q-Q scores for tumor core and invasion margin scores combined C: Q-Q scores for invasive margin scores. D: Q-Q plots for tumor core counts. E: Q-Q scores for tumor core and invasion margin counts combined F: Q-Q scores for invasive margin counts.

# Appendix 6: Machine learning predictive model assessment: three-class or two-class analysis of Immune scores


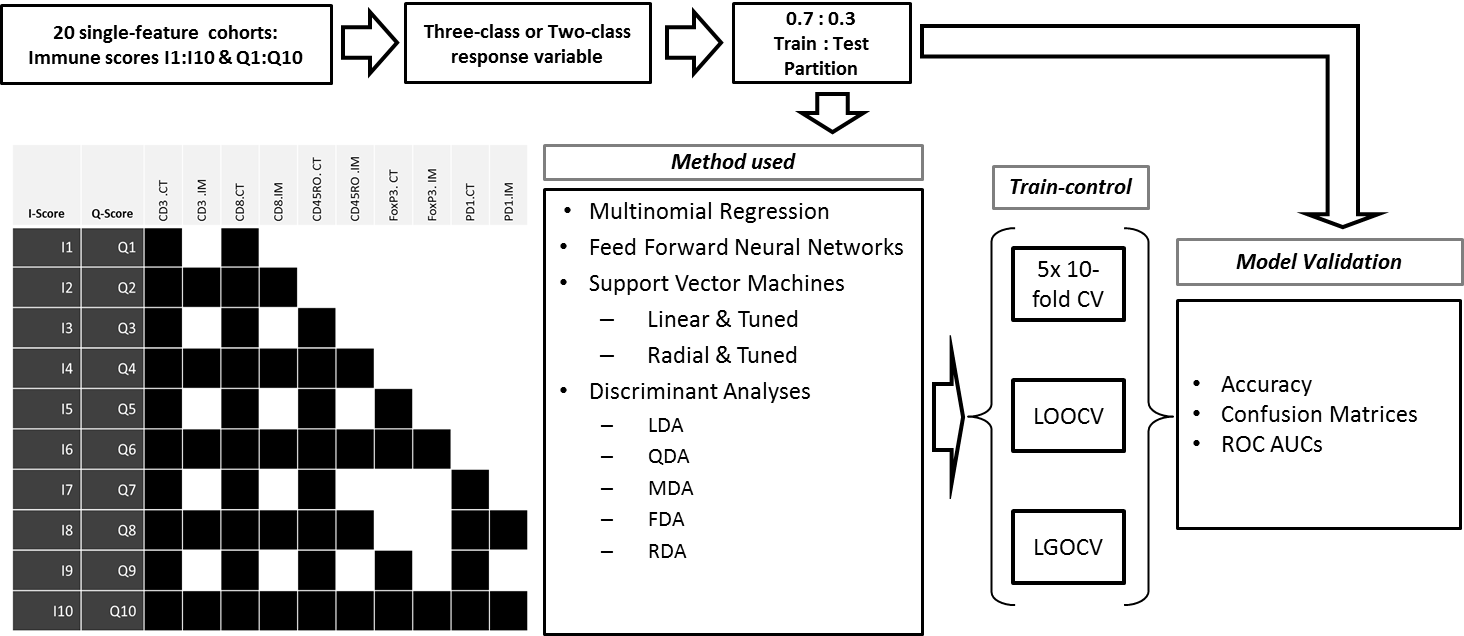


## Figure S9: Schema of machine learning model assessment for use of Immune scores as explanatory features in three-class or two-class predictions.

Immune scores are inputted as explanatory features. Data is partitioned into 70%:30% wherein 70% is used to build models by methods listed. These are tuned where appropriated and performance assessed by one of three cross-validation (CV) train controls. Models are then validated upon the 30% test data partition and assessed for their accuracy, confusion matrix classification, and Receiver Operating Characteristic (ROC) curve and associated Area Under Curve (AUC) values. **Abbreviations**: LDA: linear discriminant analysis; QDA: quadratic discriminant analysis; MDA: multiple discriminant analysis; FDA: flexible discriminant analysis; RDA: regularized discriminant analysis; CV: cross-validation, LOOCV: Leave one out cross-validation; LGOCV: Leave group out cross-validation.

# Appendix 7: Machine learning modeling overviews for three-class and two-class analysis of raw counts in selected cohorts


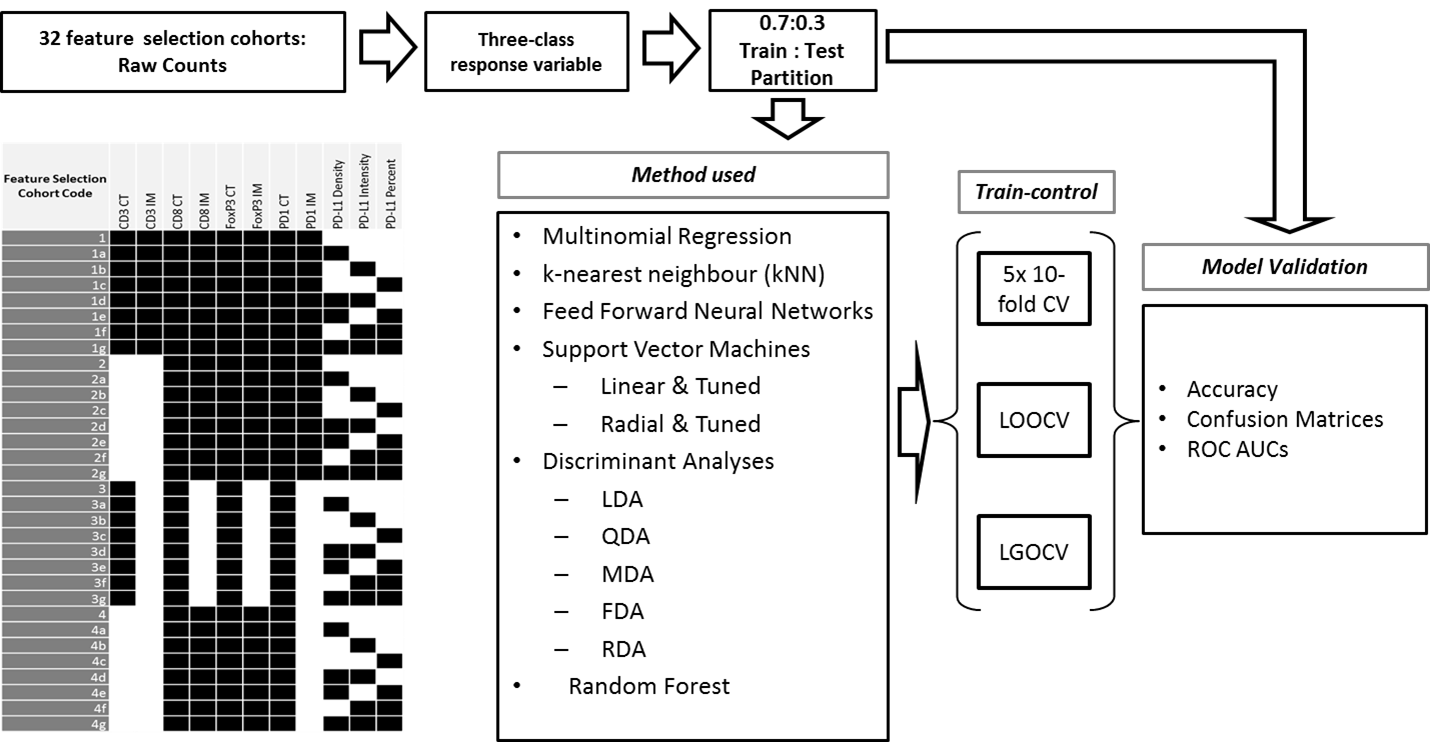


## Figure S10: Schema of machine learning model assessment for use of raw counts as independent explanatory features in three-class predictions.

Eleven independent variables populate 32 “feature selection cohorts” as explanatory features. Data is partitioned into 70%:30% wherein 70% is used to build models by methods listed. These are tuned where appropriated and performance assessed by one of three cross-validation (CV) train controls. Models are then validated upon the 30% test data partition and assessed for their accuracy, confusion matrix classification, and Receiver Operating Characteristic (ROC) curve and associated Area Under Curve (AUC) values. **Abbreviations**: LDA: linear discriminant analysis; QDA: quadratic discriminant analysis; MDA: multiple discriminant analysis; FDA: flexible discriminant analysis; RDA: regularized discriminant analysis; CV: cross-validation, LOOCV: Leave one out cross-validation; LGOCV: Leave group out cross-validation.


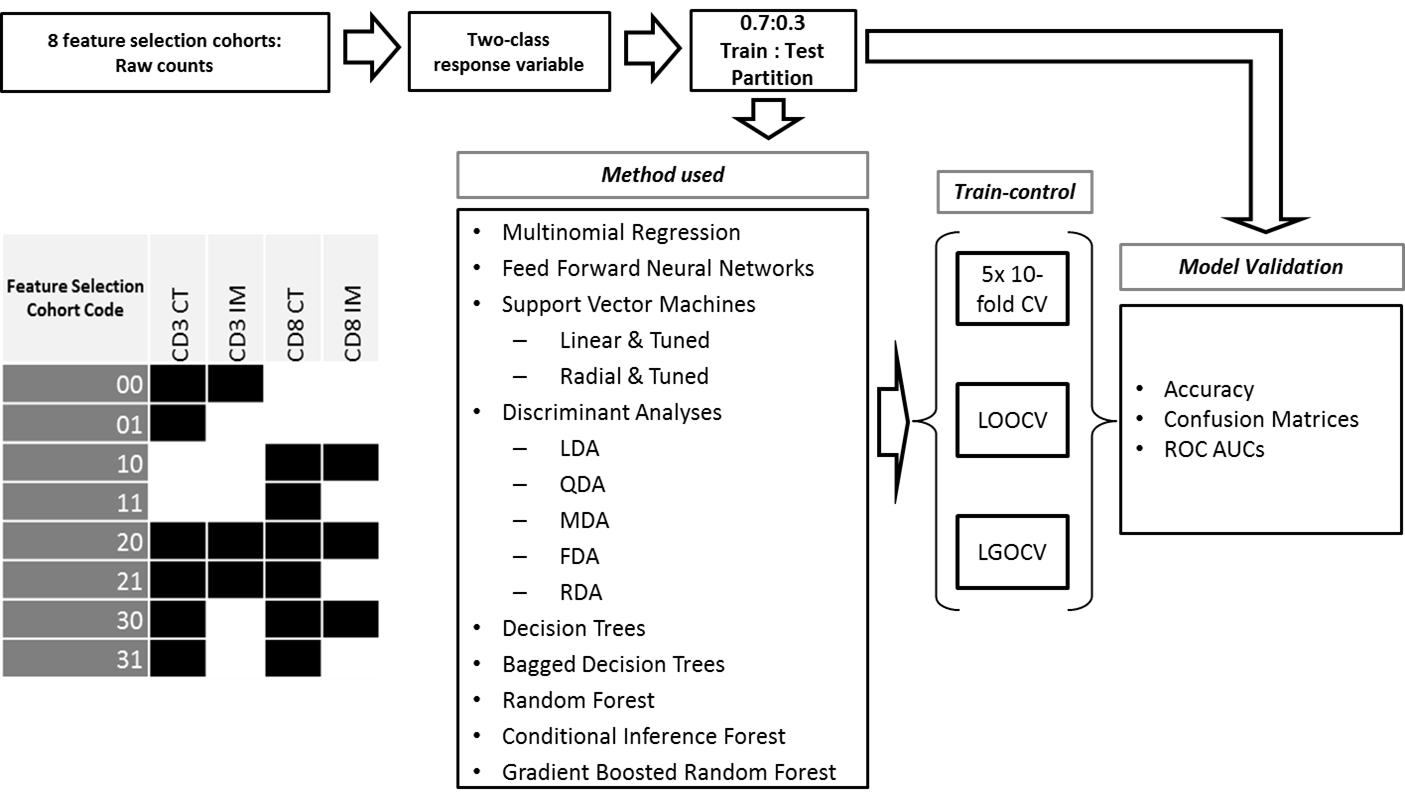


## Figure S11: Schema of machine learning model assessment for use of raw counts as independent explanatory features in two-class predictions

Four independent variables populate eight “feature selection cohorts” as explanatory features. Data is partitioned into 70%:30% wherein 70% is used to build models by methods listed. These are tuned where appropriated and performance assessed by one of three cross-validation (CV) train controls. Models are then validated upon the 30% test data partition and assessed for their accuracy, confusion matrix classification, and Receiver Operating Characteristic (ROC) curve and associated Area Under Curve (AUC) values. **Abbreviations**: LDA: linear discriminant analysis; QDA: quadratic discriminant analysis; MDA: multiple discriminant analysis; FDA: flexible discriminant analysis; RDA: regularized discriminant analysis; CV: cross-validation, LOOCV: Leave one out cross-validation; LGOCV: Leave group out cross-validation.

# Appendix 8: Key outputs from three-class machine modeling of immune scores and scores


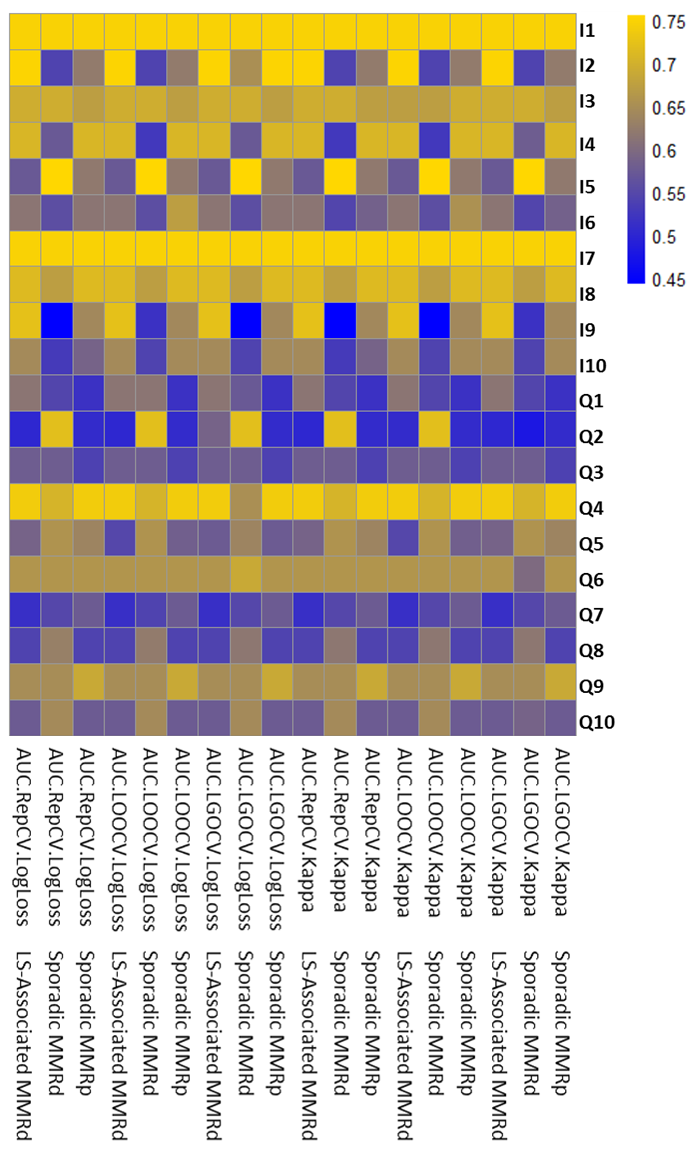


Figure S12: Heatmap of neural network multinomial logistic regression outputs (scores)

The heatmap highlights the issue of “bleed through” of the sporadic molecular groups across all three molecular groups. Quaternary score 2 performed the best; however, the high degree of misclassification makes clinically useful predicative utility dubious.


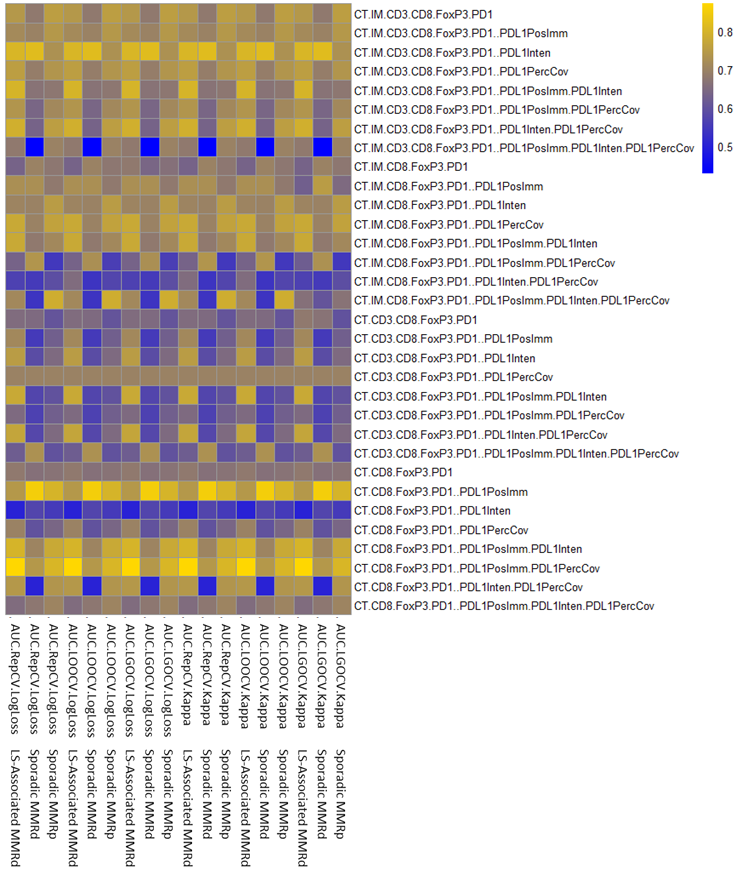


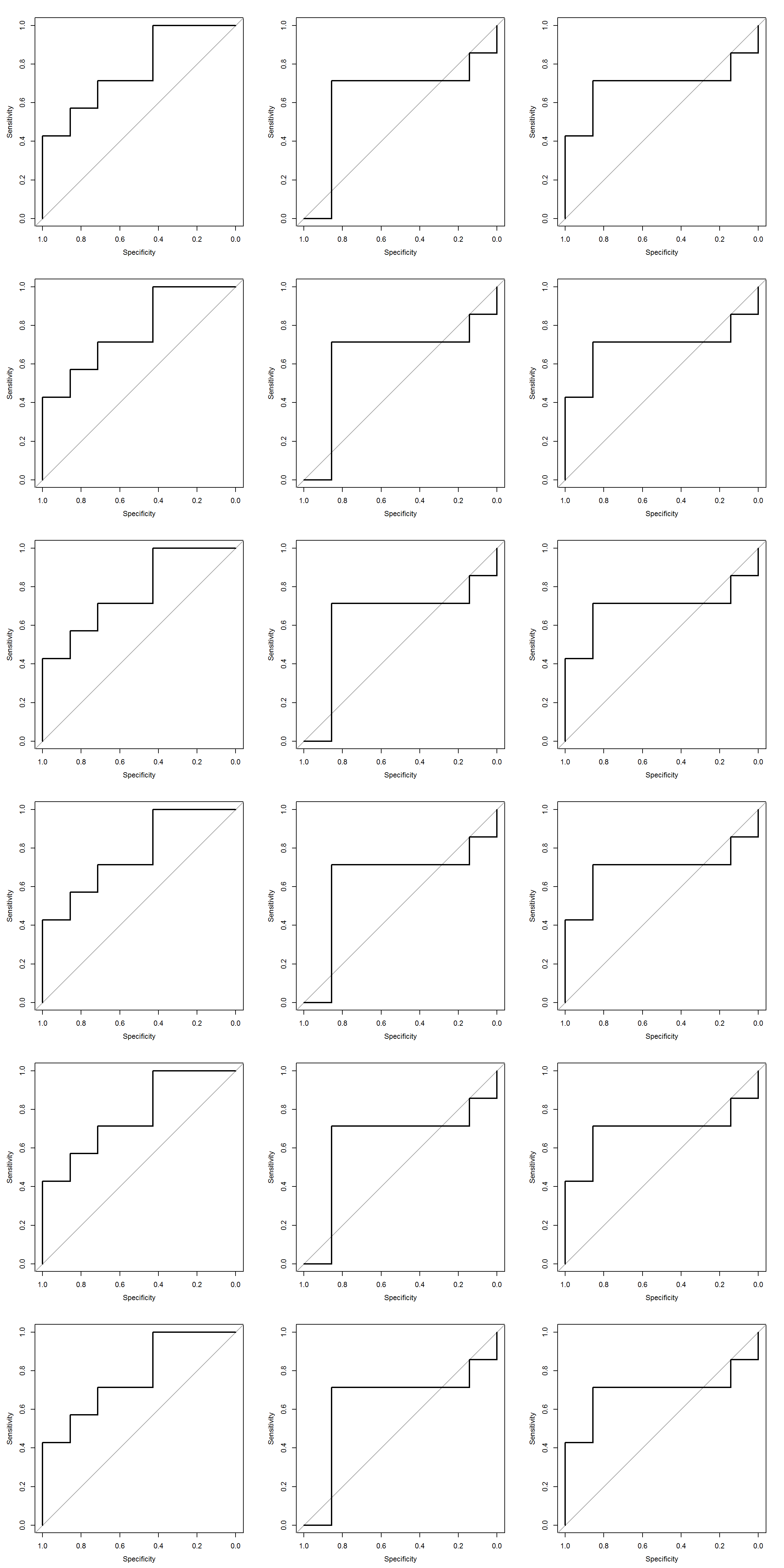


## Figure S13: Heat map of neural network multinomial logistic regression outputs (counts) with associated AUC for CD8+FOXP3+PD1+PDL1 tumor core counts

The use of counts did not improve the situation with the sporadic molecular group still mimicking both Lynch Syndrome-associated mismatch repair deficient and mismatch repair proficient tumors in the predicative modeling. This is clear from the AUC (see below heatmap) for the CD8+FOXP3+PD1+PDL1 tumor core counts. The sporadic mismatch repair deficient AUC has characteristics of both Lynch Syndrome-associated mismatch repair deficient and mismatch repair proficient tumors.


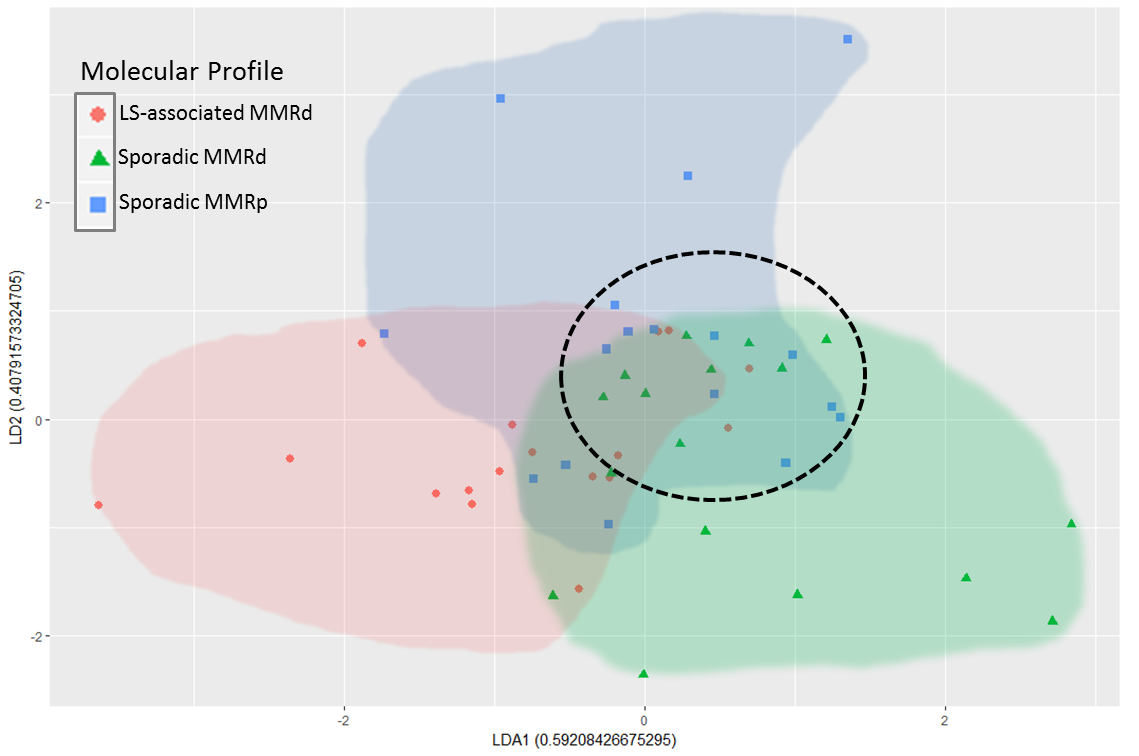


## Figure S14: Graph of Discriminant function scores for Lynch Syndrome-associated mismatch repair deficient (LS-associated MMRd), Sporadic MMRd, and Sporadic MMRp stratification.

Individual scores are plotted on the two discriminant functions wherein show little clustering of the molecular profile into discrete populations. Rather, the overlap of the three profiles (specifically Sporadic MMRd, green overlapping across segregation of Sporadic MMRp and Lynch Syndrome-associated MMRd) demonstrate the inherent phenotypic variance across the molecular profiles and the challenge of discriminating this three-class response variable.

# **Appendix 9: Machine learning modeling outputs for two-class analysis**

## Figure S15: Tabular outputs of neural network machine learning two-class analysis (mismatch repair deficient (Sporadic MMRd) vs. mismatch repair proficient (Sporadic MMRp) Immune scores).

A predictive accuracy of 0.85 was achieved for immune scores I2, Q2, Q7, and Q8.


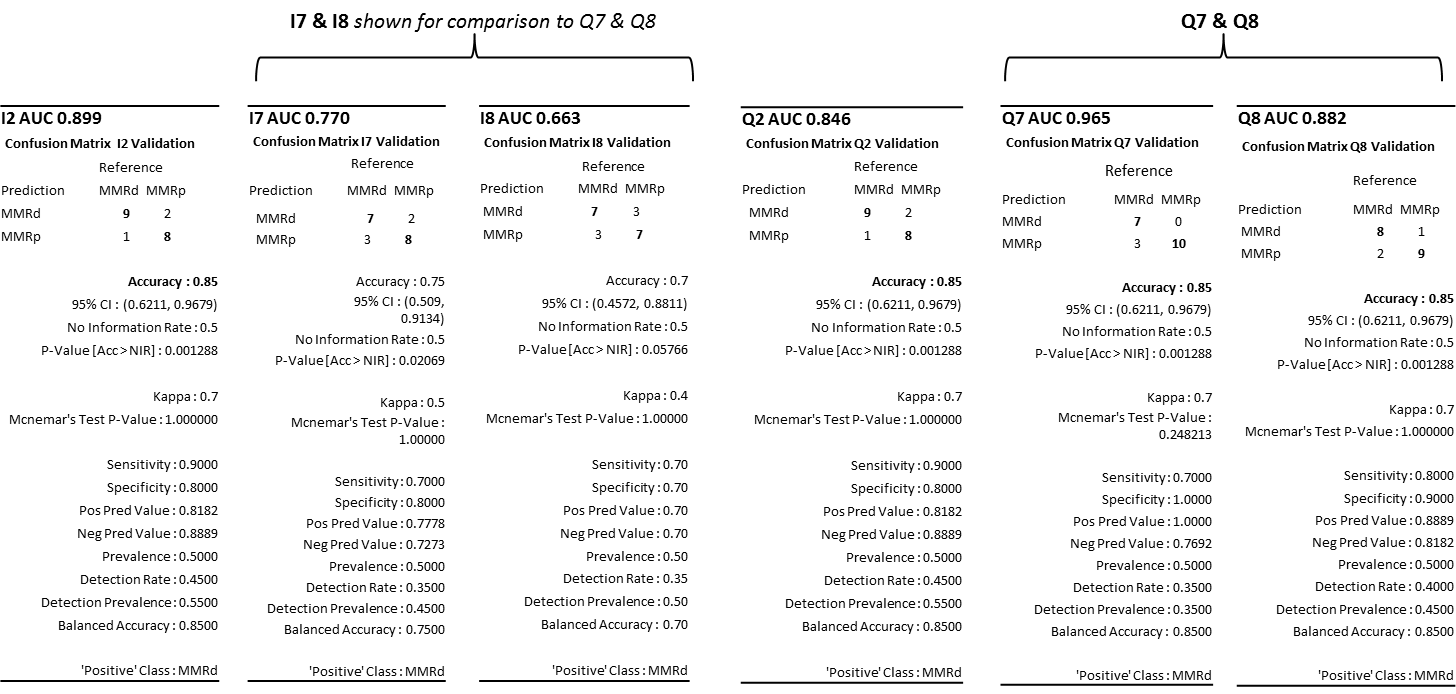


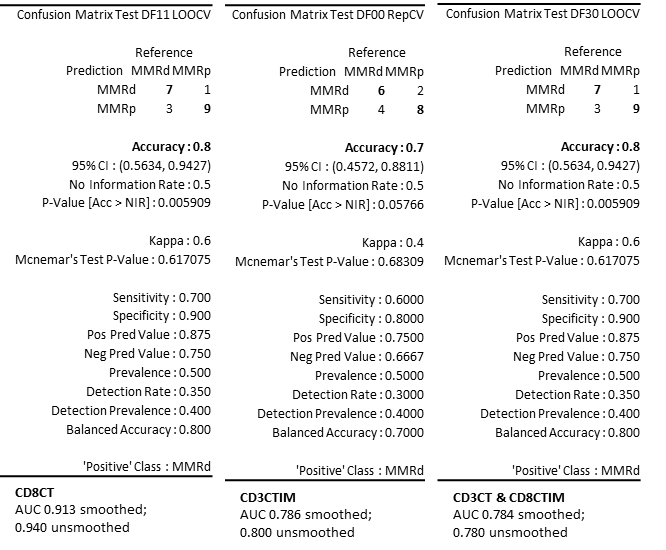


Figure S16: Tabular outputs of neural network machine learning two-class analysis (mismatch repair deficient (Sporadic MMRd) vs. mismatch repair proficient (Sporadic MMRp) counts).

A predictive accuracy of 0.80 was achieved for either a single CD8 Tumour Compartment explanatory feature; or when the three explanatory features of CD8 Tumour Compartment, CD8 Invasive Margin, and CD3 Tumour Compartment were combined**.**
